# Fascioliasis in north-central Vietnam: Assessing community knowledge, attitudes, and practices

**DOI:** 10.1371/journal.pntd.0013324

**Published:** 2025-07-21

**Authors:** Vinh Hoang Quang, Bruno Levecke, Dung Do Trung, Binh Vu Thi Lam, Le Thuy Dung, Thuy Duc Nguyen, Tran Thi Tuyen, Hien Thi Thu Nguyen, Nguyen Ngoc Ha, Brecht Devleesschauwer, Kathy Goossens, Theodorus de Jong, Linda Paredis, Nathalie De Wilde, Katja Polman, Steven Callens, Pierre Dorny, Veronique Dermauw

**Affiliations:** 1 Department of Parasitology, National Institute of Malariology, Parasitology and Entomology, Hanoi, Vietnam; 2 Department of Translational Physiology, Infectiology and Public Health, Ghent University, Merelbeke, Belgium; 3 Department of Biomedical Sciences, Institute of Tropical Medicine, Antwerp, Belgium; 4 Department of Internal Medicine and Pediatrics, Faculty of Medicine and Health Sciences, Ghent University, Ghent, Belgium; 5 Department of Epidemiology and Public Health, Sciensano, Brussels, Belgium; 6 Department of Public Health, Institute of Tropical Medicine, Antwerp, Belgium; University of Liverpool, UNITED KINGDOM OF GREAT BRITAIN AND NORTHERN IRELAND

## Abstract

**Background:**

Fascioliasis, caused by *Fasciola hepatica* and *Fasciola gigantica*, is a zoonotic disease that significantly impacts public health in agricultural communities, particularly in Vietnam. This study aims to assess the knowledge, attitudes, and practices (KAP) regarding fascioliasis among residents in a rural community in Vietnam.

**Methodology/Principal findings:**

A cross-sectional study was conducted in Dong Thanh commune, north-central Vietnam. A random sample of 621 households was selected, and 1,398 individuals participated in this study. All participants were interviewed to assess their KAP regarding fascioliasis. Household heads were also interviewed about household practices, including life cycle knowledge, health-seeking behavior, water and sanitation practices, livestock and crop management, and dietary habits. Descriptive statistics were used to assess KAP, and generalized linear models were applied to examine the associations between socio-demographic variables and KAP. Awareness of fascioliasis was low, with 85% (1,193/1,398) of respondents reporting no prior knowledge. Detailed understanding of transmission, symptoms, and prevention was limited. Only 9% (124/1,398) of participants could accurately identify the symptoms, while 12% (168/1,398) were knowledgeable about preventive measures. A high percentage of households treated drinking water (99%, 613/619), and consumption of raw vegetables was widespread, with 93% (1,083/1,168) of individuals and 95% of households reporting this practice. Males were less likely to engage in non-risky practices than females (odds ratio: 0.696; 95% confidence interval: 0.591-0.819). Most households (85%, 522/617) sourced plants from their parcels, and 67% (395/588) used animal manure as fertilizer.

**Conclusion/Significance:**

The study reveals significant gaps in KAP related to fascioliasis in Dong Thanh commune. There is a pressing need for targeted educational programs to enhance community awareness and promote safer practices to mitigate the risk of fascioliasis transmission. Future interventions should emphasize gender-specific education and broader community involvement to address these gaps effectively.

## Author summary

Fascioliasis, caused by *Fasciola hepatica* and *Fasciola gigantica*, is a zoonotic foodborne disease emerging in Vietnam. This study investigates the knowledge, attitudes, and practices (KAP) related to fascioliasis among residents of a commune in north-central Vietnam, where the disease is highly prevalent in livestock. A cross-sectional survey of 1,398 individuals and 621 households assessed life cycle knowledge, health-seeking behaviors, dietary habits, water and sanitation practices, and management of both livestock and crops. The results show that 85% of respondents were unaware of fascioliasis, with inadequate knowledge about its transmission, symptoms, and prevention. Water treatment is commonly practiced, but consumption of raw vegetables persists. Socio-demographic factors, especially gender, males more likely to engage in risky practices. The findings highlight critical gaps in KAP, emphasizing the need for targeted education to improve awareness and promote safer practices, incorporating gender-specific strategies and community engagement.

## 1. Introduction

Fascioliasis is an ubiquitous parasitic disease caused by the liver flukes *Fasciola hepatica* and *Fasciola gigantica* [1]. It primarily affects ruminants causing economic losses due to significant reductions in milk and meat production [2–5]. Fascioliasis represents a significant public health concern due to its substantial health risks to humans [6,7]. The World Health Organization (WHO) classifies fascioliasis as a neglected tropical disease, with approximately 50 million individuals infected globally and about 180 million at risk [8]. During the last decades, fascioliasis has become an emerging public health problem in many areas of the world, with an estimated annual loss of 35,000 disability adjusted life years [9]. The disease is acquired through ingesting water or plants contaminated with metacercariae, the parasite’s infective stage, which thrives in environments where human and livestock activities intersect [10].

The northern and central regions of Vietnam are highly endemic for both animal and human fascioliasis [11,12]. Traditional practices, such as the consumption of raw aquatic plants, facilitate the transmission of the disease to humans [11,13]. In a comprehensive study by De et al., which analyzed over 53,000 cases of fascioliasis in Vietnam from 1995 to 2019, a notable upward trend in the incidence of the disease was observed. The study documented a rise in the number of cases from a mere two cases reported in 1995 to a peak of 4,026 cases identified in 2019 [12].

In 2020, the General Statistics Office of Vietnam estimated the population of cattle and buffalos at 6.23 and 2.33 million, respectively [14]. In Vietnam, cattle and buffalos are typically managed by small-scale farmers, each maintaining herds of fewer than 30 animals [15], allowing them to freely graze in fields [11]. This practice significantly elevates the risk of infection among these animals, as they are exposed to contaminated environments for the majority of the year [16]. The prevalence of fascioliasis in livestock is high, especially in the Red River Delta area and in Central Vietnam, with infection rates in livestock reaching up to 72% [17].

Despite the clear public health significance of fascioliasis, there is limited information on the community knowledge, attitudes, and practices (KAP) regarding the disease in Vietnam. Understanding these factors is crucial for designing effective intervention strategies. Studies in Iran have shown that awareness and knowledge of fascioliasis are often low in affected communities, leading to behaviors that increase the risk of infection, such as the consumption of raw or improperly treated vegetables and untreated water [18]. Moreover, the utilization of livestock feces as fertilizer and the climatic conditions favoring the presence of suitable snail hosts further perpetuate the persistence and transmission of the disease [1,19]. Previous research highlighted the critical role of health education and community engagement in controlling zoonotic diseases [17,20]. Given the rising number of documented fascioliasis cases and the existing gap in comprehensive studies on the disease’s epidemiology and control in Vietnam [12], this study aims to evaluate the KAP related to fascioliasis among residents of Dong Thanh, a commune in Nghe An province (north-central Vietnam).

## 2. Methods

### 2.1. Ethics statement

This study was approved by the institutional review boards of both the National Institute of Malariology, Parasitology, and Entomology (NIMPE) in Vietnam (Approval No. 02–2022/HDDD) and the Ghent University Hospital/Faculty of Medicine and Health Sciences, Ghent University in Belgium (Approval No. BC-08915). Prior to commencing the fieldwork, the study’s aims and procedures were communicated to provincial and district health officers, as well as the Dong Thanh Commune People’s Committee, which included medical doctors and veterinarians. These local authorities subsequently granted their approval for the study. During the initial visit, detailed information about the study’s objectives and procedures was provided to all participants. Signed informed consent was obtained from each participant aged 18 years and older before they were enrolled in the study. For minors (i.e., participants between 5 and 17 years old), verbal assent was solicited and written informed consent was secured from their parents or guardians.

### 2.2. Study site

**Fig 1:**
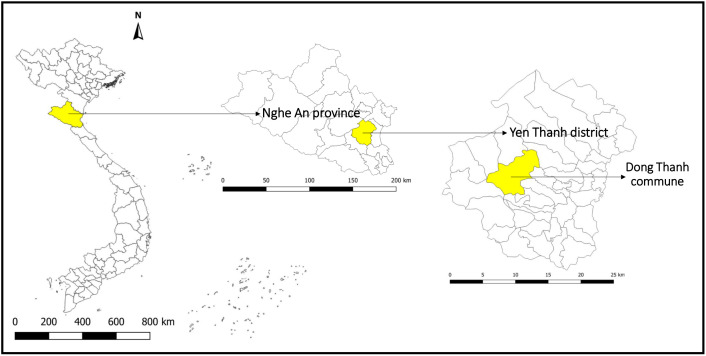
Map of Vietnam showing the locations of Nghe An province, Yen Thanh district, and Dong Thanh commune. Shapefiles were republished from the DIVA-GIS database (https://diva-gis.org/data.html) under a CC BY 4.0 license, with permission from Global Administrative Areas (GADM), original copyright 2018.

This study was conducted in Dong Thanh commune in Yen Thanh district, Nghe An province in north-central Vietnam (**Fig 1**). As of March 2022, this commune has 8,490 registered habitants across 2,036 households. The main occupation of the residents is farming. The selection of this study site was based on (i) historical data on the presence of human fascioliasis, (ii) culinary behaviors that favor disease transmission (e.g., consumption of raw vegetables), (iii) the presence of both cattle and buffalos that have access to crop fields, and (iv) the accessibility of the study site.

Nghe An province is considered one of the endemic areas for fascioliasis in Vietnam [12]. According to data of the provincial Center for Disease Control, Yen Thanh district ranked second in the province in terms of the number of fascioliasis cases, with 398 confirmed human cases reported between 2012 and 2020. Within this district, Dong Thanh commune recorded the highest number of human fascioliasis cases (39 confirmed cases during the same period), hence why the commune was selected as study site.

### 2.3. Study design and data collection

Between April and May 2022, a cross-sectional study was conducted to assess the current KAP related to fascioliasis within the Dong Thanh commune. This study was part of a multi-host study with the primary goal to estimate the prevalence of fascioliasis across all hosts in the life cycle [21]. From a list with the commune’s households provided by the local authorities, households with a minimum of three members were extracted. Subsequently, a random sample of 621 households was selected, by given each household on the list an ID number, and by using a random number sampling process in the R software [22]. During household visits, a maximum of three eligible individuals were randomly chosen to participate. In cases when the household comprised three or fewer individuals, all members were invited to participate. All selected individuals were invited to participate in the individual questionnaire. The household questionnaire was specifically administered to the household head or the member who possessed the most comprehensive knowledge of household activities.

### 2.4. Questionnaires

The individual questionnaire consisted of three comprehensive sections, each designed to capture specific aspects of participants’ KAP regarding fascioliasis (S1 Questionnaire). The first section contained questions on general demographic information, including age, gender, education level, and distance from the nearest health clinic. The second section focused on knowledge and awareness of fascioliasis, featuring questions on participants’ familiarity with the disease, its symptoms, modes of transmission, and prevention strategies. The third section explored participants’ attitudes and practices related to fascioliasis, including health-seeking behaviors, dietary habits, and the frequency of consuming raw vegetables. This section also included questions on healthcare access and reasons for not seeking formal medical treatment.

The household questionnaire was structured into four sections (S2 Questionnaire). The first section collected demographic data, including age, gender, occupation, educational attainment, marital status, and participants’ familiarity with fascioliasis. Additionally, the GPS coordinates of each household were recorded in this section. The second section encompassed 11 questions related to household water and sanitation practices. The third section, consisting of 15 questions, focused on livestock and crop management practices. The final section included 8 questions aimed at assessing culinary habits, specifically food preparation methods and the types of plants consumed.

Prior to field implementation, the research team received training on the questionnaire content, conducted pilot interviews among team members, and refined the questions to align with the local language, optimize interview duration, and enhance the clarity and comprehensibility of the phrasing for the local population. Both questionnaires were generated in English and then translated into Vietnamese. The questions were completed by interviewers using a printed version. After the field data collection, the data were entered manually into the RedCap application [23,24]. All original data are available in S1 Data.

### 2.5. Statistical data analysis

The data were exported from the RedCap application to an Excel file and consequently analysed by the statistical software R [22]. First, all responses were presented using descriptive statistics. Proportions were shown for categorical variables, whereas mean, median, and range were presented for continuous variables.

The study examined relationships between socio-demographic factors and responses from individual and household questionnaires. For each question, a new binary variable was created: responses indicating non-risky practices or correct knowledge scored 1, while risky practices or incorrect knowledge scored 0 (details in S1 and S2 Questionnaires). New variables were created to sum up the 1s and 0s, resulting in a total score for individual practices, household practices, and individual knowledge separately.

Associations between socio-demographic variables (gender, age, education and employment) and individual practice, household practice, and individual knowledge scores were analysed. For individual scores, a generalized linear mixed model with household as a random factor was used. Household practice scores were analysed using a generalized linear model. The full model (i.e., with all the socio-demographic variables included as fixed effects) was simplified by iteratively removing the least significant variable, comparing Akaike’s information criterion values to find the most parsimonious model. The significance level was set at 5%.

## 3. Results

### 3.1. Community KAP of fascioliasis in north-central Vietnam

#### 3.1.1. Socio-demographic characteristics of individual participants.

A total of 1,398 individuals were included in the study, with a slight majority of males (55.1%, 770/1,398). The age of the participants ranged from 5 to 92 years, with a mean age of 38 and a median age of 42. Farming was the predominant occupation, accounting for 59.3% (828/1,397) of respondents. School children and students made up 26.8% (374/1,397) of the population sample, while smaller proportions were workers (5.9%, 82/1,397) and government employees (3.4%, 47/1,397). In terms of education, most participants (53.9%, 753/1,398) had completed secondary school. Primary school education was the highest level achieved by 17.4% (243/1,398) of respondents, and 21.7% (304/1,398) had completed high school. A small percentage (5.2%, 73/1,398) had achieved a university degree, and a minority (1.8%, 25/1,398) had not attended school.

#### 3.1.2. Knowledge and attitudes on fascioliasis among individual participants.

Knowledge and awareness of fascioliasis among the participants were limited (**Tables A** and **B** in S1 Text). An important majority (85.4%, 1,193/1,397 had never heard of fascioliasis, while only a small fraction (13.0%, 182/1,398) recognized it as a disease affecting humans and/or livestock. Additionally, 1.6% (22/1,398) of respondents were unsure. The highest level of awareness about the disease was seen in the young adult range (18–49 years old) (99/484, 20.5%) as compared to those above 50 years old (62/558, 11.1%), and below 18 (21/355, 5.9%). Only participants who had heard about the disease or were unsure were asked further knowledge questions. Most of these participants were aware that fascioliasis impacts the liver (77.1%, 158/205). They perceived fascioliasis as somewhat serious, both at a personal (70.0%, 142/203) and national level (71.6%, 146/204). Commonly reported symptoms included abdominal pain (44.4%, 91/205) and fever (15.6%, 32/205). Female participants seemed to perceive fascioliasis more commonly as a very serious disease (31/120, 25.8%), whereas the youngest were more unsure about this (5/27, 18.5%) as compared to the other age groups.

A total of 61.5% (126/205) correctly identified fascioliasis as a parasitic infection, and 60.8% (124/204) understood that it is transmitted through contaminated plants or vegetables. Preventive measures noted included not eating raw water plants (42.4%, 87/205) and cooking vegetables (24.9%, 51/205). There was a strong belief that anyone could be infected (75.6%, 155/205), and 78.5% (161/205) were aware that specific treatment from health centers could cure the disease. Overall, knowledge levels were consistently lowest in the lowest age group (< 18 years old), as compared to the other two age groups where levels were more comparable (see **Table A** in S1 Text for more details). Information sources were primarily television (40.5%, 83/205) and health workers (33.7%, 69/205), while experience with the disease amongst direct contacts, such as neighbors (6.3%; 13/205) and household members (2.0%; 4/205) were rare.

Among 204 respondents, 58.3% (119/204) believed they could contract fascioliasis, 6.4% (13/204) believed they could not, and 35.3% (72/204) were unsure. The majority of the 205 respondents reported that they would primarily feel afraid if given a potential diagnosis (83.9%; 172/205), followed by feeling normal (4.9%; 10/205), surprise (2.0%; 4/205), and sadness or hopelessness (0.5%; 1/205). None of the respondents indicated shame, and 8.8% (18/205) were uncertain about how they would react. The youngest participants were more unsure about how they would feel (8/27, 29.6%), whereas men more commonly reported a normal reaction (6/84, 7.1%). Because of the low number of participants having responded to more than one knowledge questions (i.e., participants who had heard about fascioliasis), it was impossible to fit a model investigating the association between the socio-demographic characteristics and the individual knowledge scores.

#### 3.1.3. Practices on fascioliasis among individual participants.

A vast majority of respondents (95.9%; 1,340/1398) expressed their intention to use health facilities for general health problems, with a small fraction (3.3%; 46/1,398) preferring a pharmacy. A negligible percentage (0.1%; 2/1,398) opted to rest at home, while 3.3% (46/1,398) of the respondents were uncertain about where they would seek treatment, and this percentage was markedly higher for the youngest participants (43/355, 12.1%). The frequency of seeking health care was also examined; among 1,340 respondents, 84.6% (1,133) sought health care at least once a year. More details can be found in **Table C** in S1 Text.

**Fig 2:**
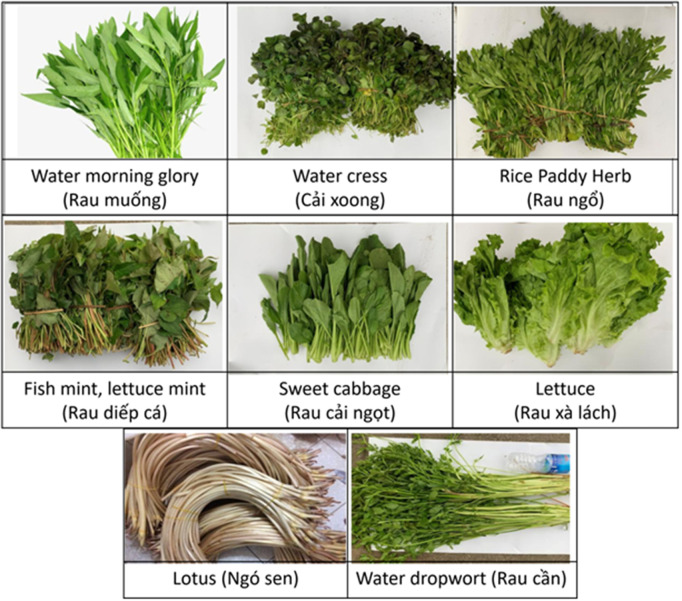
Common types of vegetables consumed raw in households in the study area.

**Fig 3:**
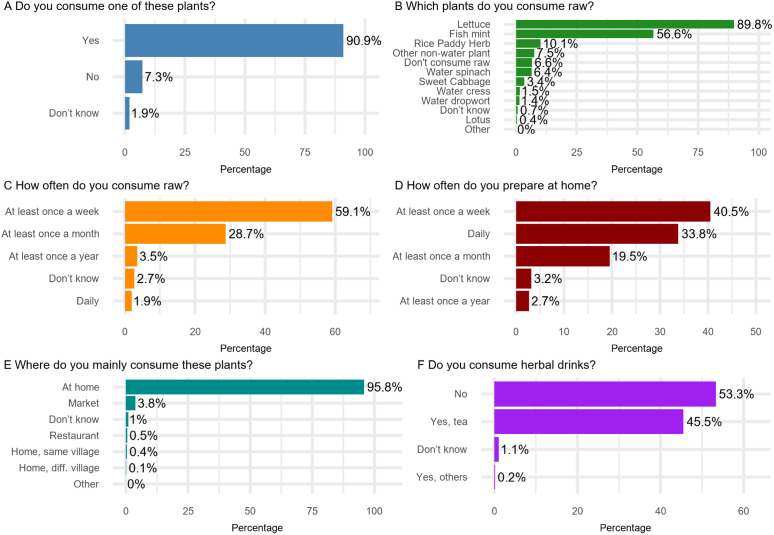
Practices related to fascioliasis among the participants in Dong Thanh commune.

Of the 1,392 respondents, 90.9% (1,265/1,392) consumed one of the specific vegetables shown to them during the interview (**Fig 2**), this percentage was lowest in the young age group (239/352, 67.9%). Of the 1,391 respondents, 83.5% (1,161/1,391) indicated they prepare these plants at home (**Fig 3**). Among the 1,168 respondents, 89.8% (1,049/1,168) consumed raw lettuce, and 56.6% (661/1,168) consumed raw fish mint, whereas a higher percentage of male participants consumed raw water spinach or water morning glory (40/478, 8.4%). Moreover, 61.0% (712/1,168) of the respondents indicated they consume these plants raw at least once a week. Regarding herbal drink consumption, out of 1,398 respondents, 45.5% (636/1,398) consumed green tea or herbal tea, while 53.3% (745/1,398) did not consume any herbal drinks, the latter percentage was higher in the youngest age group (304/355, 85.6%). Additionally, only 3.2% (45/1,398) of respondents chewed leaves, grass, or other plants found outdoors, with the lowest age group less commonly reporting this behaviour (4/355, 1.1%). When investigating the association between the individual practice scores and the socio-demographic variables, the model containing only gender was best explaining the data, with male participants having lower odds for a 1 score, indicating more risky practices, as compared to female participants (odds ratio (OR): 0.696; 95% confidence interval (95%CI): (0.591-0.819)).

### 3.2. Household practices relating to fasciolosis in north-central, Vietnam

#### 3.2.1. Socio-demographic characteristics of household participants.

A total of 621 individuals were interviewed about their household practices. Of the 620 respondents, 64.7% (401/620) were household heads, while 35.3% (219/620) were other household members. The gender distribution among the 621 participants was nearly balanced, with 50.9% (316/621) being male. The age of the participants ranged from 17 to 92 years with a mean of 50 and median age of 52 years. Farming was the primary occupation, representing 84.7% (525/620) of the surveyed individuals. Other occupations included workers (6.9%; 43/620), civil servants or government cadres (3.9%; 24/620), others (4.5%; 28/620), and miscellaneous professions (3.9%). Among the 621 individuals surveyed, education levels varied, ranging from no formal schooling (0.5%; 3/621) to university training (4.7%; 29/621). The largest group (65.7%; 408/621) had completed secondary school, followed by high school graduates (26.1%; 162/621), while 3.1% (19/621) had finished primary education. Details about the socio-demographic characteristics can be found in **Table C** in S1 Text.

#### 3.2.2. Water and sanitation practices in households.

**Fig 4:**
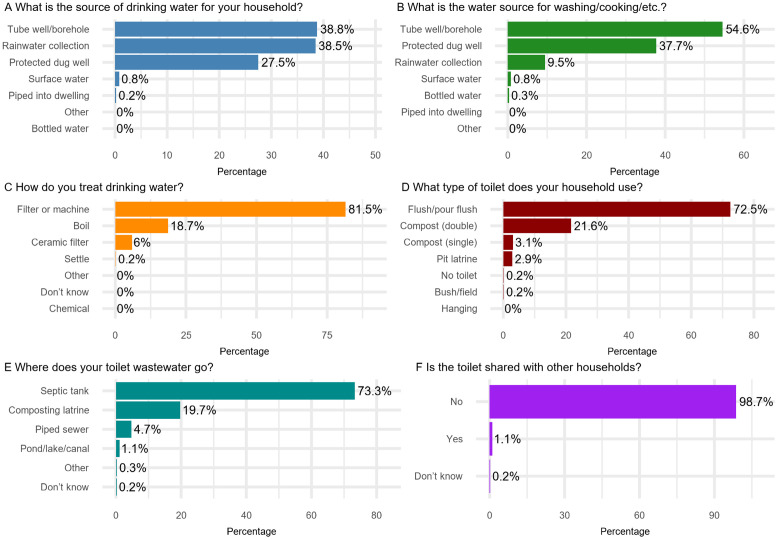
Household water and sanitation practices in Dong Thanh commune.

Water and sanitation practices are described in **Fig 4**. Households primarily relied on tube wells/boreholes (241/621, 38.8%) and rainwater collection (239/621, 38.5%) for their drinking water, while tube wells/boreholes (339/621, 54.6%) and protected dug wells (234/621, 37.7%) were water sources used for other purposes in the households. Nearly all households (613/619, 98.7%) treated their drinking water, with the majority using filters or water machines (501/615, 81.5%). Most households (450/621, 72.5%) had access to flush/pour flush toilets, and the wastewater was predominantly managed through septic tanks (454/619, 73.3%). Additionally, a vast majority of households (609/617, 98.7%) had private toilet facilities. More details are provided in **Table D** in S1 Text.

**Fig 5:**
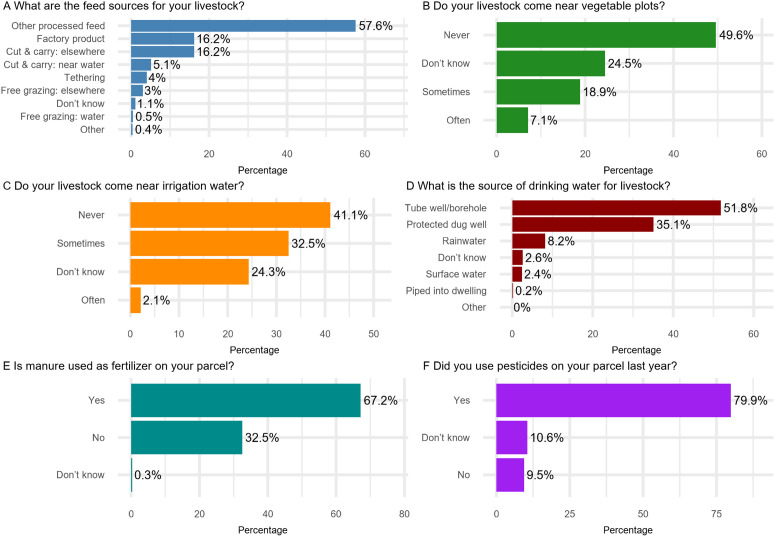
Crop and livestock management practices of households in the Dong Thanh commune.

Related to livestock and crop management (**Fig 5**), most households (588/621, 94.7%) owned agricultural parcels, with a significant portion (411/585, 70.3%) being actively engaged in farming activities. These parcels were predominantly used for crop cultivation, particularly rice, which was the primary crop for most households (565/587, 96.3%). Livestock ownership was prevalent, with 567/584 households (96.4%) owning livestock. In terms of livestock interactions with water bodies used for vegetable cultivation, 40/567 respondents (7.1%) reported that their livestock frequently accessed these areas, while 107/567 (18.9%) indicated occasional access. Conversely, 281/567 respondents (49.6%) stated that their livestock never came into contact with such water bodies. Furthermore, 139/567 respondents (24.5%) were uncertain about their livestock’s interaction with these water sources. Among these, 119/567 households (21.0%) owned cattle, and 78/567 households (13.8%) owned buffalo. Manure was commonly utilized as fertilizer (395/588, 67.2%), with composting being the preferred treatment method of animal feces (388/395, 98.2% of those using manure). Pesticide use was widespread among households (469/587, 79.9%), indicating a reliance on chemical inputs for crop management. More details can be found in **Table E** in S1 Text.

**Fig 6:**
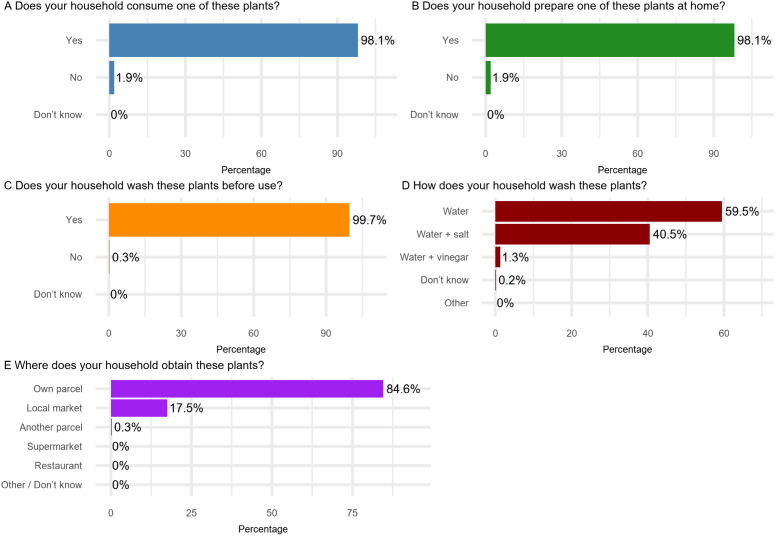
Culinary practices among the households in the Dong Thanh commune.

**Fig 6** illustrates the culinary practices among households in the Dong Thanh commune. Most households engaged in the consumption and preparation of various plants. Nearly all households (98.1%; 608/620) consumed these plants, and an equal percentage prepare them at home. Commonly consumed raw plants included lettuce (92.1%; 569/617), fish mint/lettuce mint (64.6%; 399/617), and rice paddy herb (13.4%; 83/617), while the most frequently cooked plants were water spinach/water morning glory (95.3%; 588/617) and sweet cabbage (85.6%; 528/617). Washing practices were prevalent, with 99.7% (616/617) of households washing these plants before use, primarily with water alone (59.5%; 367/617) or with water and salt (40.5%; 250/617). Only 1.3% (8/617) washed the plants with vinegar and water. Most households (84.6%; 522/617) obtained these plants from their own parcels, while a smaller percentage (17.5%; 108/617) purchased them from local markets. None of the investigated socio-demographic variables was found to be significantly associated with the household practice scores. For more details, see **Table F** in S1 Text.

## 4. Discussion

Fascioliasis, caused by *F. hepatica* and *F. gigantica*, significantly impacts the health of both humans and animals, mainly as the result of liver damage. This study provides crucial insights into the KAP related to fascioliasis among residents of a rural community in the north-central coast region of Vietnam. Dong Thanh commune is representative of many fascioliasis-endemic rural communities in north-central Vietnam. This representativeness is based on shared ecological, agricultural, and behavioral characteristics such as livestock grazing in crop fields, the frequent consumption of raw vegetables, and the presence of freshwater bodies favorable for intermediate hosts. Furthermore, surveillance data confirm that Dong Thanh has one of the highest reported fascioliasis case numbers in Yen Thanh district—one of the most affected districts in Nghe An province. These factors support the generalizability of our findings to other similar endemic settings within Vietnam.

### Lack of knowledge about fascioliasis

A substantial knowledge deficit regarding fascioliasis was revealed in our study, with about 85.3% of participants indicating no prior awareness of the disease. This awareness level is considerably lower compared to findings from other regions. For example, a study in northern Vietnam reported that only 11.5% of participants had never heard of liver fluke [13], consistent with results from Central Vietnam [17,25,26]. Another study by Lugong et al., (2024) in the Philippines reported a higher level of awareness, with 81.6% of participants indicating they had heard of fascioliasis [27]. A detailed understanding of fascioliasis’s transmission, symptoms, and prevention among our respondents was notably deficient. This aligns with findings from regions where fascioliasis was prevalent but underreported, indicating a global need for enhanced public health education [1].

### Common practices of consuming raw vegetables and misconceptions about safe handling practices

Our study reveals that the consumption of raw vegetables was frequently practiced within the community. Of the 1,168 respondents, 1,083 (92.7%) indicated consumption of at least one type of raw vegetable. Additionally, 712 participants (61.8%) noted consuming raw vegetables at a minimum frequency of once per week. These findings align with a study by Phi et al. in northern Vietnam, where 91.2% reported consuming raw vegetables [13]. Conversely, Quy et al.‘s research found lower rates in central Vietnam, with consumption ranging from 28.2% to 33.8% [17].

In Vietnam, there is a common belief that washing raw vegetables with clean water or soaking them in a saltwater or vinegar solution after washing, makes them safe to eat. Our results showed that 99.8% of households wash vegetables, 59.5% using water, 40.5% saltwater, and 1.3% vinegar water. However, Phuong’s study demonstrated that washing vegetables, whether by rinsing them under running water or scrubbing them by hand, was largely ineffective at removing metacercariae, with less than 2% of the larvae being detached [28]. Additionally, exposure to a 5% acetic acid solution, comparable to commercial vinegar, resulted in 93.6% to 96.0% of metacercariae surviving after less than 20 minutes of treatment. Similarly, soaking vegetables in saltwater for less than 20 minutes failed to effectively kill or detach the metacercariae [28]. These findings are further corroborated by Hassan et al. (2008), who reported that acetic acid solutions below 5% were ineffective at killing *Fasciola* metacercariae, even after 30 minutes of exposure [29].

### Modifying livestock and crop farming practices to reduce fascioliasis transmission

During the KAP study, we concurrently investigated the prevalence of *Fasciola* infection in hosts within the same area. The results revealed a notable discrepancy: animal (buffalo and cattle) infection rates were 51.5% by copro-microscopy and 54.1% by antibody ELISA, while human infection rates were 0% by copro-microscopy and only 0.07% by antibody ELISA [6]. At the same time, this KAP study showed that conditions appear highly conducive to the potential transmission of *Fasciola*. Indeed, this is due to the widespread practice of free-grazing buffalo and cattle in the commune, and people frequently consume raw vegetables, including aquatic plants.

This low infection rate in humans could be explained by several key observations. Firstly, among the raw vegetables consumed, non-aquatic varieties such as lettuce (92.1%) and herbs (64.6%) were the most preferred. Secondly, 84.6% of the vegetables consumed were sourced from household gardens, which included some aquatic plants like fish mint, morning glory, and rice paddy herb. Additionally, households primarily used agricultural land (including submerged land) for rice cultivation (96.3%), with significant portions also allocated to corn (30.7%) and potatoes/sweet potatoes (14.7%). Only a small fraction (3.2%) of the land was dedicated to the cultivation of aquatic vegetables. Furthermore, the study found that out of 395 households using livestock manure in agriculture, 388 households (98.2%) composted the manure before use. In Dong Thanh commune, animal manure is typically collected and piled for 3–6 months before being used in agriculture. Composting livestock manure before use helps reduce the risk of *Fasciola* transmission. A study by Moazeni et al. (2010) demonstrated that composting at high temperatures and for a sufficient duration effectively inactivates *Fasciola* eggs and reduces the formation of miracidia [30].

The infection rate of *Fasciola* in the livestock population remains very high, exceeding 50%. This can be attributed to the practice of free grazing of livestock near agricultural fields, which is a significant risk factor for *Fasciola* transmission. Free grazing allows cattle to consume contaminated water and aquatic plants, simultaneously dispersing feces into the agriculture fields. This aligns with previous research by Nguyen et al., which indicated a high risk of infection in livestock due to extensive grazing practices [16].

In general, the practices observed in Dong Thanh commune appear to present significant risks for fascioliasis transmission, particularly due to the high prevalence of infection in livestock, widespread free-grazing, and the consumption of raw vegetables. Combined with the low level of knowledge about the disease, this creates an apparently “dangerous” situation. However, the accompanying prevalence study shows that human infection rates remain very low [21], suggesting that transmission to humans is occurring at a minimal level. Upon closer examination, certain practices, such as the composting of livestock manure, may play a role in reducing the survival of *Fasciola* eggs and limiting transmission risks. Additionally, the practice of growing vegetables in household gardens, with a preference for non-aquatic varieties, may further mitigate potential exposure. Nonetheless, further research is required to gain deeper insights into the specific factors that influence the transmission dynamics and to determine which practices are most effective in reducing the risk of fascioliasis transmission to humans.

## Conclusion

This study reveals significant gaps in KAP related to fascioliasis among residents of Dong Thanh commune, highlighting a substantial lack of awareness. Risky behaviors, such as the frequent consumption of raw vegetables in humans, and free grazing of animals in fields, are prevalent. Although the current prevalence in humans in the area is reported to be low, it is key to stay alert about the potential for transmission, suggesting that certain practices, such as manure composting and the preference for non-aquatic vegetables, may help mitigate transmission. To reduce the risk of fascioliasis transmission, targeted interventions are required. These should include educational programs aimed at raising community awareness about the disease and promoting safer practices in livestock management and vegetable consumption. The role of household gardens in limiting exposure should also be emphasized, alongside improved composting techniques to ensure the inactivation of *Fasciola* eggs. It is essential to incorporate gender-specific strategies and community involvement to enhance the effectiveness of these interventions. Further investigations should elucidate the factors hampering transmission to humans.

## Supporting information

S1 Questionnaire Individual questionnaire for all participants.
(DOCX)

S2 Questionnaire Household questionnaire for head of household.
(DOCX)

S1 Data Original data. (XLSX)

S1 Text Data analysis results. (DOCX)

## References

[1] Mas-Coma S, Bargues MD, Valero MA. Fascioliasis and other plant-borne trematode zoonoses. International Journal for Parasitology. 2005;35(11–12):1255–78. doi: 10.1016/j.ijpara.2005.07.010 16150452

[2] Charlier J, Duchateau L, Claerebout E, Williams D, Vercruysse J. Associations between anti-Fasciola hepatica antibody levels in bulk-tank milk samples and production parameters in dairy herds. Preventive Veterinary Medicine. 2007;78(1):57–66. doi: 10.1016/j.prevetmed.2006.09.010 17095109

[3] Schweizer G, Braun U, Deplazes P, Torgerson PR. Estimating the financial losses due to bovine fasciolosis in Switzerland. Veterinary Record. 2005;157(7):188–93. 16100368 10.1136/vr.157.7.188

[4] Rioux M-C, Carmona C, Acosta D, Ward B, Ndao M, Gibbs BF, et al. Discovery and validation of serum biomarkers expressed over the first twelve weeks of Fasciola hepatica infection in sheep. International Journal for Parasitology. 2008;38(1):123–36. doi: 10.1016/j.ijpara.2007.07.017 17888928 PMC7094367

[5] Copeman D, Copland R, Gray GD. Overcoming liver fluke as a constraint to ruminant production in South-East Asia. Australian Centre for International Agricultural Research. 2008.

[6] Mas-Coma S. Epidemiology of fascioliasis in human endemic areas. Journal of helminthology. 2005;79(3):207–16. 16153314 10.1079/joh2005296

[7] Mas-Coma S. The importance of emerging and re-emerging zoonotic diseases: recognition, monitoring and control. In: Odongo NE, Garcia M, Viljoen GJ. 2010;277.

[8] Nyindo M, Lukambagire A-H. Fascioliasis: An Ongoing Zoonotic Trematode Infection. BioMed Research International. 2015;2015:1–8. doi: 10.1155/2015/786195 PMC4568335 26417603

[9] Fürst T, Keiser J, Utzinger J. Global burden of human food-borne trematodiasis: a systematic review and meta-analysis. The Lancet Infectious Diseases. 2012;12(3):210–21. doi: 10.1016/s1473-3099(11)70294-8 22108757

[10] Cwiklinski K, O’neill S, Donnelly S, Dalton J. A prospective view of animal and human Fasciolosis. Parasite immunology. 2016;38(9):558–68. 27314903 10.1111/pim.12343 PMC5053257

[11] Bui TD, Doanh PN, Saegerman C, Losson B. Current status of fasciolosis in Vietnam: an update and perspectives. J Helminthol. 2015;90(5):511–22. doi: 10.1017/s0022149x15000929 26564097

[12] De NV, Minh PN, Le TH, Dung DT, Duong TT, Tuan BV, et al. A multidisciplinary analysis of over 53,000 fascioliasis patients along the 1995–2019 countrywide spread in Vietnam defines a new epidemiological baseline for One Health approaches. One Health. 2024;19:100869. doi: 10.1016/j.onehlt.2024.100869 39220760 PMC11364005

[13] Phi NTT, Nguyen TTB, Le TTH, Do DT, Lenaerts M, Losson B, et al. Foodborne zoonotic trematode infections in Yen Bai, Vietnam: a situational analysis on knowledge, attitude, and practice (KAP) and risk behaviors. J Prev Med Hyg. 2022;63(2):E310–e9. doi: 10.15167/2421-4248/jpmh2022.63.2.2104 35968061 PMC9351405

[14] Office GS. Statistical summary book of Vietnam. Statistical Publishing House. 2020.

[15] Geurden T, Somers R, Thanh NTG, Vien LV, Nga VT, Giang HH, et al. Parasitic infections in dairy cattle around Hanoi, northern Vietnam. Veterinary Parasitology. 2008;153(3–4):384–8. doi: 10.1016/j.vetpar.2008.01.031 18328629

[16] Lan Anh NT, Thanh DTH, Hoan DH, Thuy DT, Khong NV, Anderson N. The transmission of Fasciola spp. to cattle and contamination of grazing areas with Fasciola eggs in the Red River Delta region of Vietnam. Trop Anim Health Prod. 2014;46(4):691–6. doi: 10.1007/s11250-014-0542-4 24510222

[17] Quy T, Yeatman H, Flood VM, Chuong N, Tuan B. Prevalence and risks of fascioliasis among adult cohorts in Binh Dinh and Quang Ngai provinces-central Viet Nam. 2015.

[18] Sazmand A, Alipoor G, Zafari S, Zolhavarieh SM, Alanazi AD, Sargison ND. Assessment of knowledge, attitudes and practices relating to parasitic diseases and anthelmintic resistance among livestock farmers in Hamedan, Iran. Frontiers in veterinary science. 2020;7:584323. doi: 10.3389/fvets.2020.584323 33195608 PMC7649137

[19] Nguyen TGT, Le TH, Dao THT, Tran TLH, Praet N, Speybroeck N, et al. Bovine fasciolosis in the human fasciolosis hyperendemic Binh Dinh province in Central Vietnam. Acta Tropica. 2011;117(1):19–22. doi: 10.1016/j.actatropica.2010.09.003 20920452

[20] Francisco I, Jiz M, Rosenbaum M, Baltazar P, Steele JA. Knowledge, attitudes and practices related to schistosomiasis transmission and control in Leyte, Philippines. PLoS Negl Trop Dis. 2019;13(5):e0007358. doi: 10.1371/journal.pntd.0007358 PMC6516667 31048882

[21] Hoang VQ, Dermauw V, Do DT, Vu BTL, Nguyen HTT, Nguyen HN. Prevalence and risk factors related to Fasciola spp. transmission in Northern Vietnam: A cross-sectional study in multiple hosts. medRxiv. 2024;2024. doi: 10.24310192

[22] R Core Team. R: A Language and Environment for Statistical Computing. Vienna, Austria: R Foundation for Statistical Computing. 2024.

[23] Harris PA, Taylor R, Thielke R, Payne J, Gonzalez N, Conde JG. A metadata-driven methodology and workflow process for providing translational research informatics support. J Biomed Inform. 2009;42(2):377–81. doi: 10.1016/j.jbi.2008.08.010 18929686 PMC2700030

[24] Harris PA, Taylor R, Minor BL, Elliott V, Fernandez M, O’Neal L, et al. The REDCap consortium: building an international community of software platform partners. Journal of biomedical informatics. 2019;95:103208. doi: 10.1016/j.jbi.2019.103208 31078660 PMC7254481

[25] Nguyen V, Nguyen V, Bui V, Tran M. Trial model of fascioliasis control in two communes of Phu Cat district, Binh Dinh province. Vietnam Journal of Practical Medicine. 2011;796:152–6.

[26] Nguyen TH. Study on some characteristics and treatment effectiveness of fascioliasis with triclabendazole in Tinh Ky and Nghia Son communes, Quang Ngai province. Ha Noi: National Institute of Malariology‐Parasitology and Entomology. 2012.

[27] Lugong R, Nicolas K, Bermudez J. Knowledge, attitude and practices of small-hold cattle and carabao owners towards fasciolosis in Northeastern Luzon, Philippines. Journal of Veterinary Medicine. 2023.

[28] Nguyen THXP. Foodborne zoonotic trematode infections and integrated control in Vietnam: aspects related to snails and vegetables. 2022.

[29] Hassan AAA, Shoukary NM, El-Motayam M, Morsy ATA. Efficacy of five chemicals on *Fasciola* gigantica encysted metacercariae infectivity. J Egypt Soc Parasitol. 2008;38(3):919–28. 19209774

[30] Moazeni M, Ansari-Lari M, Masoodfar M, Hosseinzadeh S, Mootabi Alavi A. Lethal effect of high temperatures on the eggs of Fasciola hepatica. Iranian Journal of Veterinary Research. 2010;11(2):168–73.

