# Supplementary material for: Fascioliasis in north-central Vietnam: Assessing community knowledge, attitudes, and practices
Source: PLoS Negl Trop Dis. 2025 Jul 21;19(7):e0013324. doi: 10.1371/journal.pntd.0013324 (PMC12313056; doi:10.1371/journal.pntd.0013324)
Supplement: S1 Questionnaire — Individual questionnaire for all participants. (DOCX) [file pntd.0013324.s001.docx]

# **Info S1**_**Individual Questionnaire for Human Participants**

# INDIVIDUAL QUESTIONNAIRE

|  | Village | | Household | | | Individual |
| --- | --- | --- | --- | --- | --- | --- |
| ***Individual code*** |  |  |  |  |  |  |

**Part 1: General information**

| 1 | Date (DD/MM/YYYY) |
| --- | --- |
|  | _____/____/_______ |
| 3 | Gender *(circle correct)* |
|  | M / F |
| 4 | Age (in years) |
|  | ______ |
| 5 | Occupation |
|  | School children/student |
|  | Farmer |
|  | Worker |
|  | Cadre, government, civil servant |
|  | Other, specify_________________________________ |
| 6 | Education |
|  | Primary school |
|  | Secondary school |
|  | High school |
|  | University or higher |
|  | Did not go to school |
| 7 | Marital status |
|  | Married |
|  | Single |
|  | Widowed |
|  | Other, specify__________________________________ |
| 8 | How far do you live from the nearest health clinic or hospital? |
|  | 0-10 kilometers |
|  | 11-20 kilometers |
|  | 21-30 kilometers |
|  | More than 30 kilometers |
|  | Don’t know |

**Part 2: Knowledge & awareness**

| Q1 | Have you ever heard about fascioliasis? | Risk score |  |
| --- | --- | --- | --- |
|  | No | 0 | >>Q16 |
|  | Yes, as a human disease | 1 | >>Q2 |
|  | Yes, as a disease in livestock | 1 | >>Q2 |
|  | Yes, as a disease in both humans & livestock | 1 | >>Q2 |
|  | Don’t know | 0 | >>Q2 |

| Q2 | What is fascioliasis? *(multiple choices possible)* | Risk score |  |
| --- | --- | --- | --- |
|  | A disease affecting skin | 0 | >>Q3 |
|  | A disease affecting liver | 1 | >>Q3 |
|  | A disease affecting bones | 0 | >>Q3 |
|  | A disease affecting heart | 0 | >>Q3 |
|  | Other, specify__________________ | 0 | >>Q3 |
|  | Don’t know | 0 | >>Q3 |

| Q3 | In your opinion, how serious a disease is fascioliasis? |  |  |
| --- | --- | --- | --- |
|  | Very serious |  | >>Q4 |
|  | Somewhat serious |  | >>Q4 |
|  | Not very serious |  | >>Q4 |
|  | Don’t know |  | >>Q4 |

| Q4 | In your opinion, how serious a problem is fascioliasis in your country? |  |
| --- | --- | --- |
|  | Very serious | >>Q5 |
|  | Somewhat serious | >>Q5 |
|  | Not very serious | >>Q5 |
|  | Don’t know | >>Q5 |

| Q5 | What symptoms does fascioliasis cause? *(multiple choices possible)* | Risk score |  |
| --- | --- | --- | --- |
|  | Headache | 0 | >>Q6 |
|  | Epilepsy | 0 | >>Q6 |
|  | Blurry vision | 0 | >>Q6 |
|  | Backache | 0 | >>Q6 |
|  | Abdominal pain | 1 | >>Q6 |
|  | Fever | 1 | >>Q6 |
|  | Itch | 0 | >>Q6 |
|  | Rash on skin | 0 | >>Q6 |
|  | Jaundice | 1 |  |
|  | Other, specify: _________________________ | N/A | >>Q6 |
|  | Don’t know | 0 | >>Q6 |

| Q6 | What agent is causing fascioliasis? *(multiple choices possible)* | Risk score |  |
| --- | --- | --- | --- |
|  | Bad weather | 0 | >>Q8 |
|  | Lack of nutritious food | 0 | >>Q8 |
|  | You are born with it | 0 | >>Q8 |
|  | A virus | 0 | >>Q7 |
|  | A parasite | 1 | >>Q7 |
|  | A bacteria | 0 | >>Q7 |
|  | Other, specify: ___________________ | 0 | >>Q7 |
|  | Don’t know | 0 | >>Q7 |

| Q7 | How can a person get fascioliasis? *(multiple choices possible)* | Risk score |  |
| --- | --- | --- | --- |
|  | Through consumption infected organs | 0 | >>Q8 |
|  | Through consumption contaminated plants/vegetables | 1 | >>Q8 |
|  | Through consumption infected meat | 0 | >>Q8 |
|  | Through drinking alcohol | 0 | >>Q8 |
|  | Through consumption of infected fish | 0 | >>Q8 |
|  | Contact with animals | 0 |  |
|  | Other, specify:__________________________ | N/A | >>Q8 |
|  | Don’t know | 0 | >>Q8 |

| Q8 | How can human fascioliasis be prevented? *(multiple choices possible)* | Risk score |  |
| --- | --- | --- | --- |
|  | Isolating infected livestock | 0 | >>Q9 |
|  | Isolating infected humans | 0 | >>Q9 |
|  | Washing water plants | 1 | >>Q9 |
|  | Do not eat raw water plants | 1 | >>Q9 |
|  | Cooking meat or organs | 0 | >>Q9 |
|  | Cooking vegetables \| Nấu chín rau | 1 | >>Q9 |
|  | Treatment of humans | 1 | >>Q9 |
|  | Treatment of animals | 1 | >>Q9 |
|  | It cannot be prevented | 0 | >>Q9 |
|  | Other, specify:___________________ | N/A | >>Q9 |
|  | Don’t know | N/A | >>Q9 |

| Q9 | In your opinion, who can be infected with human fascioliasis? *(multiple choices possible)* | Risk score |  |
| --- | --- | --- | --- |
|  | Only children | 0 | >>Q10 |
|  | Only poor people | 0 | >>Q10 |
|  | Only homeless people | 0 | >>Q10 |
|  | Only elderly people | 0 | >>Q10 |
|  | Only men | 0 | >>Q10 |
|  | Only women | 0 | >>Q10 |
|  | Anybody | 1 | >>Q10 |
|  | Others, specify:___________________ | 1 | >>Q10 |
|  | Don’t know | 0 | >>Q10 |

| Q10 | Can fascioliasis be cured? *(multiple choices possible)* | Risk score |  |
| --- | --- | --- | --- |
|  | Yes, with herbal medicine | 0 | >>Q11 |
|  | Yes, home rest without medicine | 0 | >>Q11 |
|  | Yes, specific treatment given by health centre | 1 | >>Q11 |
|  | Yes, using another method, specify:___________________ | N/A | >>Q11 |
|  | It cannot be cured | 0 | >>Q11 |
|  | Don’t know | 0 | >>Q11 |

| Q11 | Where did you hear about fascioliasis?  *(multiple choices possible)* |  |  |
| --- | --- | --- | --- |
|  | School |  | >>Q12 |
|  | Internet |  | >>Q12 |
|  | Newspapers and magazines |  | >>Q12 |
|  | Radio |  | >>Q12 |
|  | TV |  | >>Q12 |
|  | Billboards/posters |  | >>Q12 |
|  | Health workers |  | >>Q12 |
|  | Family, friends, neighbours and colleagues |  | >>Q12 |
|  | Other, specify:__________________________ |  | >>Q12 |
|  | Don’t know |  | >>Q12 |

| Q12 | Do you know people who have/had fascioliasis?  *(multiple choices possible)* |  |
| --- | --- | --- |
|  | Yes, I have/had fascioliasis | >>Q13 |
|  | Yes, household members | >>Q14 |
|  | Yes, relatives (not from household) | >>Q14 |
|  | Yes, neighbours | >>Q14 |
|  | Yes, friends or colleagues | >>Q14 |
|  | Yes, other people, specify:__________________________ | >>Q14 |
|  | No | >>Q14 |
|  | Don’t know | >>Q14 |

| Q13 | If you have or had fascioliasis, at what point did you go to the health facility? |  |  |
| --- | --- | --- | --- |
|  | When treatment on my own didn’t work |  | >>Q14 |
|  | As soon as I realized that my symptoms might be related with fascioliasis |  | >>Q14 |
|  | As soon as I was feeling ill |  | >>Q14 |
|  | I did not go to the health facility |  | >>Q14 |
|  | Other, specify:__________________________ |  | >>Q14 |
|  | Don’t know |  | >>Q14 |

**Part 3: Attitudes & practices**

| Q14 | Do you think you could get fascioliasis? |  |  |
| --- | --- | --- | --- |
|  | Yes, because ______________________________________________________________________________________________ |  | >>Q15 |
|  | No, because ______________________________________________________________________________________________ |  | >>Q15 |
|  | Don’t know |  | >>Q15 |

| Q15 | What would be your reaction if you were to found out that you have fascioliasis? *(multiple choices possible)* |  |
| --- | --- | --- |
|  | Normal |  |
|  | Fear | >>Q16 |
|  | Surprise | >>Q16 |
|  | Sadness or hopelessness | >>Q16 |
|  | Shame | >>Q16 |
|  | Other, specify___________________________________ | >>Q16 |
|  | Don’t know | >>Q16 |

| Q16 | Where do you usually go if you are sick, or to treat a general health problem? *(multiple choices possible)* |  |
| --- | --- | --- |
|  | Go to health facility | >>Q18 |
|  | Go to pharmacy | >>Q17 |
|  | Go to traditional healer | >>Q17 |
|  | Pursue other self-treatment options | >>Q17 |
|  | Nowhere, rest at home | >>Q17 |
|  | Other, specify___________________________________ | >>Q17 |
|  | Don’t know | >>Q17 |

| Q17 | If you would not go to the health facility, what is the reason? *(multiple choices possible)* |  |
| --- | --- | --- |
|  | No need |  |
|  | Not sure where to go | >>Q19 |
|  | Cost | >>Q19 |
|  | Difficulties with transportation/distance to clinic | >>Q19 |
|  | Do not trust medical workers | >>Q19 |
|  | Do not like attitude of medical workers | >>Q19 |
|  | Cannot leave work (overlapping work hours with medical facility working hours) | >>Q19 |
|  | Do not want to find out something is really wrong | >>Q19 |
|  | Other, specify___________________________________ | >>Q19 |
|  | Don’t know | >>Q19 |

| Q18 | How often do you generally seek health care at a clinic or hospital |  |
| --- | --- | --- |
|  | Twice a year or more | >>Q19 |
|  | Once per year | >>Q19 |
|  | Less than once a year | >>Q19 |
|  | Don’t know | >>Q19 |

| Q19 | Do you consume one of these plants? *(you will be shown some pictures)* |  |  |
| --- | --- | --- | --- |
|  | Yes |  | >>Q20 |
|  | No |  | >>Q20 |
|  | Don’t know |  | >>Q20 |

| Q20 | Do you prepare one of these plants at home? *(you will be shown some pictures)* | Risk score |  |
| --- | --- | --- | --- |
|  | Yes | 0 | >>Q21 |
|  | No | 1 | >>Q25 |
|  | Don’t know | N/A | >>END |

| Q21 | Which of the following plants do you consume raw? *(you will be shown some pictures)(multiple choices possible)* | Risk score |  |
| --- | --- | --- | --- |
|  | Water spinach, water morning glory (Rau muống) | 0 | >>Q22 |
|  | Water cress (Cải xoong) | 0 | >>Q22 |
|  | [Rice Paddy Herb](https://web.archive.org/web/20140908201208/http:/www.uni-graz.at/~katzer/engl/Limn_aro.html) (Rau ngổ) | 0 | >>Q22 |
|  | Salad (Rau xà lách) | 1 | >>Q22 |
|  | Sweet Cabbage (Rau cải ngọt) | 1 | >>Q22 |
|  | Lotus (Ngó sen) | 0 | >>Q22 |
|  | Fish mint, lettuce mint (Rau diếp cá) | 0 | >>Q22 |
|  | Water dropwort (Rau cần) | 0 | >>Q22 |
|  | Other non-water plant | 1 |  |
|  | Others, specify:_____________________________ | 1 | >>Q22 |
|  | I don’t consume raw plants/vegetables | 1 | >>Q22 |
|  | Don’t know | N/A | >>Q22 |

| Q22 | If you consume these raw, how often? (last year) |  |  |
| --- | --- | --- | --- |
|  | Daily |  | >>Q23 |
|  | At least once a week |  | >>Q23 |
|  | At least once a month |  | >>Q23 |
|  | At least once a year |  | >>Q23 |
|  | Don’t know |  | >>Q23 |

| Q23 | If you prepare these at home (either raw or cooked), how often? (last year) |  |  |
| --- | --- | --- | --- |
|  | Daily |  | >>Q24 |
|  | At least once a week |  | >>Q24 |
|  | At least once a month |  | >>Q24 |
|  | At least once a year |  | >>Q24 |
|  | Don’t know |  | >>Q24 |
|  |  |  |  |

| Q24 | Where do you mainly consume these plants? |  |  |
| --- | --- | --- | --- |
|  | At home |  | >>Q25 |
|  | At home of other people in same village |  | >>Q25 |
|  | At home of other people in different village |  | >>Q25 |
|  | At the market |  | >>Q25 |
|  | In a restaurant |  | >>Q25 |
|  | Others, specify:_______________________ |  | >>Q25 |
|  | Don’t know |  | >>Q25 |

| Q25 | Do you ever consume herbal drinks? | Risk score |  |
| --- | --- | --- | --- |
|  | Yes, tea | 0 | >>Q26 |
|  | Yes, others: specify:____________________ | 0 | >>Q26 |
|  | No | 1 | >>Q26 |
|  | Don’t know | N/A | >>Q26 |

| Q26 | Do you ever chew on leaves, grass, or other plants you find outdoors? | Risk score |  |
| --- | --- | --- | --- |
|  | Yes | 0 | >>Q25 |
|  | No | 1 | >>Q25 |
|  | Don’t know | N/A | >>Q25 |

| Q27 | Do you ever consume water chestnut? | Risk score |  |
| --- | --- | --- | --- |
|  | Yes | 1 | >>END |
|  | No | 0 | >>END |
|  | Don’t know | N/A | >>END |

Thank you for your participation!
