# Supplementary material for: Fascioliasis in north-central Vietnam: Assessing community knowledge, attitudes, and practices
Source: PLoS Negl Trop Dis. 2025 Jul 21;19(7):e0013324. doi: 10.1371/journal.pntd.0013324 (PMC12313056; doi:10.1371/journal.pntd.0013324)
Supplement: S2 Questionnaire — Household questionnaire for head of household. (DOCX) [file pntd.0013324.s002.docx]

# **Info S2**_**Household Questionnaire for Human Participants**

**HOUSEHOLD QUESTIONNAIRE**

|  | Village | | Household | | |
| --- | --- | --- | --- | --- | --- |
| ***HH code*** |  |  |  |  |  |

**Part 1 General information**

| 1 | Date (DD/MM/YYYY) |
| --- | --- |
|  | _____/____/_______ |
| 2 | Gender *(circle correct)* |
|  | M / F |
| 3 | Age (in years) |
|  | ______ |
| 4 | Role in household |
|  | Head of household |
|  | Other_______________ |

**Part 2 Water & sanitation practices**

| Q1 | What is the source of drinking-water for members of your household? *(indicate most important one(s))* |  | Risk score |
| --- | --- | --- | --- |
|  | Piped water into dwelling | >>Q2 | 1 |
|  | Tube well/borehole | >>Q2 | 1 |
|  | Protected dug well | >>Q2 | 1 |
|  | Rainwater collection | >>Q2 | 1 |
|  | Bottled water | >>Q2 | 1 |
|  | Surface water (river, dam, lake, pond, stream, canal, irrigation channels) | >>Q1A | 0 |
|  | Other, specify_________________________ | >>Q2 | 0 |
|  | Don’t know | >>Q2 | N/A |

| Q1A | Specify the type of surface water (for drinking)? |  |
| --- | --- | --- |
|  | River | >>Q2B |
|  | Dam | >>Q2B |
|  | Lake | >>Q2B |
|  | Pond, within premises | >>Q2B |
|  | Pond, outside premises | >>Q2B |
|  | Stream | >>Q2B |
|  | Canal | >>Q2B |
|  | Irrigation channels, within premises | >>Q2B |
|  | Irrigation channels, outside premises | >>Q2B |
|  | Mountain water | >>Q2B |
|  | Other, specify:____________________________ | >>Q2B |
|  | Don’t know | >>Q2B |

| Q2 | What is the source of water used by your household for other purposes, such as washing vegetables, cooking and hand washing? *(indicate most important one(s))* |  | Risk score |
| --- | --- | --- | --- |
|  | Piped water into dwelling | >>Q3 | 1 |
|  | Tube well/borehole | >>Q3 | 1 |
|  | Protected dug well | >>Q3 | 1 |
|  | Rainwater collection | >>Q3 | 1 |
|  | Bottled water | >>Q3 | 1 |
|  | Surface water (river, dam, lake, pond, stream, canal, irrigation channels) | >>Q2A | 0 |
|  | Other, specify_____________________ | >>Q3 | N/A |
|  | Don’t know | >>Q3 | N/A |

| Q2A | Specify the type of surface water (for other purposes)? |  |
| --- | --- | --- |
|  | River | >>Q2B |
|  | Dam | >>Q2B |
|  | Lake | >>Q2B |
|  | Pond, within premises | >>Q2B |
|  | Pond, outside premises | >>Q2B |
|  | Stream | >>Q2B |
|  | Canal | >>Q2B |
|  | Irrigation channels, within premises | >>Q2B |
|  | Irrigation channels, outside premises | >>Q2B |
|  | Mountain water | >>Q2B |
|  | Other, specify:____________________________ | >>Q2B |
|  | Don’t know | >>Q2B |

| Q2B | Do livestock have access to this surface water? |  | Risk score |
| --- | --- | --- | --- |
|  | Usually | >>Q3 | 0 |
|  | Often | >>Q3 | 0 |
|  | Sometimes | >>Q3 | 0 |
|  | Never | >>Q3 | 1 |
|  | Don’t know | >>Q3 | N/A |

| Q3 | Do you treat your water in any way to make it safer to drink |  | Risk score |
| --- | --- | --- | --- |
|  | Yes | >>Q4 | 1 |
|  | No | >>Q5 | 0 |
|  | Don’t know | >>Q5 | N/A |

| Q4 | How do you treat the water to make it safer to drink? *(indicate the most important ones)* |  |
| --- | --- | --- |
|  | Boil | >>Q5 |
|  | Using a chemical | >>Q5 |
|  | Use filter, water machine | >>Q5 |
|  | Let it stand and settle | >>Q5 |
|  | Other (specify_____________________) | >>Q5 |
|  | Don’t know | >>Q5 |

| Q5 | What kind of toilet facility do members of your household use? *(indicate most important one(s))* |  | Risk score |
| --- | --- | --- | --- |
|  | Flush/pour flush | >>Q6 | 1 |
|  | Composting toilet (single tank) | >>Q6 | 1 |
|  | Composting toilet (double tanks) | >>Q6 | 1 |
|  | Hanging toilet/hanging latrine/‘Pond toilet’ | >>Q7 | 0 |
|  | Dug pit latrine | >>Q6 | 0 |
|  | Bush or field | >>Q9 | 0 |
|  | Other (specify ) | >>Q6 | N/A |
|  | Don’t know | >>Q9 | N/A |
|  | No toilet | >>Q9 | 0 |

| Q6 | Where does wastewater from your toilet go? *(indicate most important one(s))* |  | Risk score |
| --- | --- | --- | --- |
|  | Piped sewer system | >>Q7 | 1 |
|  | Septic tank | >>Q7 | 1 |
|  | Pond/lake/canal | >>Q7 | 0 |
|  | Composting latrine | >>Q7 | 1 |
|  | Other, specify: ____________________ | >>Q7 | 0 |
|  | Don’t know | >>Q7 | N/A |

| Q7 | Do you share this facility with other households? |  |
| --- | --- | --- |
|  | Yes | >>Q8 |
|  | No | >>Q9 |
|  | Don’t know | >>Q9 |

| Q8 | How many households use this toilet facility? |  |
| --- | --- | --- |
|  | Other households share this toilet, number: __________________ | >>Q9 |
|  | Any member of the public can use this toilet | >>Q9 |
|  | Don’t know | >>Q9 |

**Part 3 Livestock & crop management**

| Q9 | Do you or any of your household currently own any agriculture parcels exclusively or jointly with someone else? |  |
| --- | --- | --- |
|  | Yes | >>Q10 |
|  | No | >>Q9A |
|  | Don’t know | >>Q9A |

| Q9A | Do you or any of your household work on agriculture parcels? |  |
| --- | --- | --- |
|  | Yes | >>Q10 |
|  | No | >>Q22 |
|  | Don’t know | >>Q22 |

| Q10 | What is the primary use of this parcel? |  |
| --- | --- | --- |
|  | Livestock | >>Q12 |
|  | Crops | >>Q11 |
|  | Livestock and crops | >>Q11 |

| Q11 | What crops are cultivated on this parcel? *(multiple choices are possible)* |  |
| --- | --- | --- |
|  | Rice | >>Q12 |
|  | Water plants | >>Q12 |
|  | Sweet potato/potato | >>Q12 |
|  | Flower | >>Q12 |
|  | Groundnuts | >>Q12 |
|  | Corn | >>Q12 |
|  | Bean | >>Q12 |
|  | Fruit |  |
|  | Other: _______________________________ | >>Q12 |
|  | Don’t know | >>Q12 |

| Q12 | Do you or any of your household currently own any livestock exclusively or jointly with someone else? |  |
| --- | --- | --- |
|  | Yes | >>Q13 |
|  | No | >>Q20 |
|  | Don’t know | >>Q20 |

| Q13 | Which livestock do you own? *(multiple choices are possible)* |  |
| --- | --- | --- |
|  | Cattle | >>Q14 |
|  | Buffalo | >>Q14 |
|  | Goat | >>Q14 |
|  | Pigs | >>Q14 |
|  | Horse | >>Q14 |
|  | Poultry | >>Q14 |
|  | Other, specify:___________________________ | >>Q14 |
|  | Don’t know | >>Q14 |

| Q14 | How many livestock do all members of your household own? *(multiple entries are possible)* |  |
| --- | --- | --- |
|  | Cattle: _________________________ | >>Q15 |
|  | Buffalo : _________________________ | >>Q15 |
|  | Goat: : _________________________ | >>Q15 |
|  | Pigs: _________________________ | >>Q15 |
|  | Horse: _________________________ | >>Q15 |
|  | Poultry: _________________________ | >>Q15 |
|  | Other: _____________________________ | >>Q15 |

| Q15 | What are the feed sources for your livestock? *(indicate most important one(s))* |  | Risk Score |
| --- | --- | --- | --- |
|  | Cut and carry, grown in or near waterbodies | >>Q16 | 0 |
|  | Cut and carry, grown elsewhere | >>Q16 | 1 |
|  | Tethering | >>Q16 | 1 |
|  | Factory product | >>Q16 | 1 |
|  | Free roaming/Grazing, in or near waterbodies | >>Q16 | 0 |
|  | Free roaming/Grazing, elsewhere | >>Q16 | 1 |
|  | Other processed feed source |  | 1 |
|  | Other, specify: ___________________ | >>Q16 | N/A |
|  | Don’t know | >>Q16 | N/A |

| Q16 | What is the purpose of your livestock? *(indicate most important one(s))* |  |
| --- | --- | --- |
|  | Dairy | >>Q17 |
|  | Meat | >>Q17 |
|  | Skin | >>Q17 |
|  | Draft power | >>Q17 |
|  | Sale |  |
|  | Other, specify:___________________ | >>Q17 |
|  | Don’t know | >>Q17 |

| Q17 | Do your livestock come in or near waterbodies where vegetables for human consumption are being grown? |  | Risk Score |
| --- | --- | --- | --- |
|  | Often | >> Q18 | 0 |
|  | Sometimes | >> Q18 | 0 |
|  | Never | >> Q18 | 1 |
|  | Don’t know | >> Q18 | N/A |

| Q18 | Do your livestock come in or near water supply for crop water irrigation? |  | Risk Score |
| --- | --- | --- | --- |
|  | Often | >> Q19 | 0 |
|  | Sometimes | >> Q19 | 0 |
|  | Never | >> Q19 | 1 |
|  | Don’t know | >> Q19 | N/A |

| Q19 | What is the source of drinking-water for your livestock? *(indicate most important one(s))* |  | Risk score |
| --- | --- | --- | --- |
|  | Piped water into dwelling | >>Q20 | 1 |
|  | Tube well/borehole | >>Q20 | 1 |
|  | Protected dug well | >>Q20 | 1 |
|  | Rainwater collection | >>Q20 | 1 |
|  | Surface water (river, dam, lake, pond, stream, canal, irrigation channels) | >>Q20 | 0 |
|  | Other, specify___________________________ | >>Q20 | N/A |
|  | Don’t know | >>Q20 | N/A |

| Q20 | Do you use manure of cattle/buffalo/goat/horse/pig/human as a fertilizer of your parcel? |  | Risk Score |
| --- | --- | --- | --- |
|  | Yes | >>Q20A | 0 |
|  | No | >>Q21 | 1 |
|  | Don’t know | >>Q21 | N/A |

| Q20A | How do you treat manure before use as fertilizer? |  | Risk score |
| --- | --- | --- | --- |
|  | No treatment (use fresh manure) | >>Q21 | 0 |
|  | Composting before use | >>Q21 | 1 |
|  | Others, specify___________________________ | >>Q21 | N/A |
|  | Don’t know | >>Q21 | N/A |

| Q21 | Did you use pesticides on your parcel the last year? |  |
| --- | --- | --- |
|  | Yes, specify type and frequency used the last year: _____________________ | >>Q22 |
|  | No | >>Q22 |
|  | Don’t know_____________________ | >>Q22 |

**Part 4 Culinary practices**

| Q22 | Who is mainly responsible for cooking in this household? |  |
| --- | --- | --- |
|  | Grandmother/father | >>Q23 |
|  | Mother | >>Q23 |
|  | Father | >>Q23 |
|  | Children | >>Q23 |
|  | Others, specify:______________________________ | >>Q23 |
|  | Don’t know | >>Q23 |

| Q23 | Does this household consume one of these plants? *(you will be shown some pictures)* |  | Risk score |
| --- | --- | --- | --- |
|  | Yes | >>Q24 | 0 |
|  | No | >>Q24 | 1 |
|  | Don’t know | >>Q24 | N/A |

| Q24 | Does this household prepare one of these plants at home (either raw or cooked)? *(you will be shown some pictures)* |  | Risk Score |
| --- | --- | --- | --- |
|  | Yes | >>Q25 | 0 |
|  | No | >>END | 1 |
|  | Don’t know | >>END | N/A |

| Q25 | Which of the following plants does your household consume raw? *(you will be shown some pictures)(multiple choices possible)* |  |
| --- | --- | --- |
|  | Water spinach, water morning glory (Rau muống) | >>Q26 |
|  | Water cress (Cải xoong) | >>Q26 |
|  | [Rice Paddy Herb](https://web.archive.org/web/20140908201208/http:/www.uni-graz.at/~katzer/engl/Limn_aro.html) (Rau ngổ) | >>Q26 |
|  | Salad (Rau xà lách) | >>Q26 |
|  | Sweet Cabbage (Rau cải ngọt) | >>Q26 |
|  | Lotus (Ngó sen) | >>Q26 |
|  | Fish mint, lettuce mint (Rau diếp cá) | >>Q26 |
|  | Water dropwort (Rau cần) | >>Q26 |
|  | Other non-waterplants |  |
|  | Others, specify:_____________________________ | >>Q26 |
|  | My household does not consume raw plants/vegetables | >>Q26 |
|  | Don’t know | >>Q26 |

| Q26 | Which of the following plants does your household consume cooked? *(you will be shown some pictures)(multiple choices possible)* |  |
| --- | --- | --- |
|  | Water spinach, water morning glory (Rau muống) | >>Q27 |
|  | Water cress (Cải xoong) | >>Q27 |
|  | [Rice Paddy Herb](https://web.archive.org/web/20140908201208/http:/www.uni-graz.at/~katzer/engl/Limn_aro.html) (Rau ngổ) | >>Q27 |
|  | Salad (Rau xà lách) | >>Q27 |
|  | Sweet Cabbage (Rau cải ngọt) | >>Q27 |
|  | Lotus (Ngó sen) | >>Q27 |
|  | Fish mint, lettuce mint (Rau diếp cá) | >>Q27 |
|  | Water dropwort (Rau cần) | >>Q27 |
|  | Other non-waterplants |  |
|  | Others, specify:_____________________________ | >>Q27 |
|  | My household does not consume cooked plants/vegetables | >>Q27 |
|  | Don’t know | >>Q27 |

| Q27 | Does your household wash these plants before use? |  | Risk score |
| --- | --- | --- | --- |
|  | Yes | >>Q28 | 1 |
|  | No | >>Q29 | 0 |
|  | Don’t know | >>Q28 | N/A |

| Q28 | How does your household wash these plants? *(indicate the most important one(s))* |  | Risk score |
| --- | --- | --- | --- |
|  | With water | >>Q29 | 0 |
|  | With water + vinegar | >>Q29 | 1 |
|  | With water + salt | >>Q29 | 0 |
|  | Other, specify____________________ | >>Q29 | N/A |
|  | Don’t know | >>Q29 | 0 |

| Q29 | Where does your household obtain these plants *(indicate the most important one(s))* |  |
| --- | --- | --- |
|  | Local market (Wet market) | >>END |
|  | Cultivated from own parcel | >>END |
|  | Cultivated from other parcel (e.g. family and friends | >>END |
|  | Super market | >>END |
|  | Restaurant | >>END |
|  | Other, specify____________________ | >>END |
|  | Don’t know | >>END |

Thank you for your participation!
