# Supplementary material for: Fascioliasis in north-central Vietnam: Assessing community knowledge, attitudes, and practices
Source: PLoS Negl Trop Dis. 2025 Jul 21;19(7):e0013324. doi: 10.1371/journal.pntd.0013324 (PMC12313056; doi:10.1371/journal.pntd.0013324)
Supplement: S1 Text — (DOCX) [file pntd.0013324.s004.docx]

## S4 Text - Data analysis results

## Community knowledge, attitudes, and practices of fascioliasis in north-central Vietnam

**Table A. Participant knowledge & awareness of fascioliasis among participants in the Dong Thanh commune.**

| **Question** | **Total** | | **Gender** | | | | **Age (years)** | | | | | |
| --- | --- | --- | --- | --- | --- | --- | --- | --- | --- | --- | --- | --- |
|  |  |  | **Male** | | **Female** | | **<18** | | **18-49** | | **>=50** | |
|  | **n** | **%** | **n** | **%** | **n** | **%** | **n** | **%** | **n** | **%** | **n** | **%** |
| **Have you ever heard about fascioliasis?** | **N= 1,397** |  | **N= 626** |  | **N= 771** |  | **N= 355** |  | **N= 484** |  | **N= 558** |  |
| No | 1,193 | 85.4 | 542 | 86.6 | 651 | 84.4 | 328 | 92.4 | 379 | 78.3 | 486 | 87.1 |
| Yes, as a human disease | 66 | 4.7 | 22 | 3.5 | 44 | 5.7 | 10 | 2.8 | 32 | 6.6 | 24 | 4.3 |
| Yes, as a disease in livestock | 17 | 1.2 | 8 | 1.3 | 9 | 1.2 | 5 | 1.4 | 9 | 1.9 | 3 | 0.5 |
| Yes, as a disease in both humans & livestock | 99 | 7.1 | 44 | 7.0 | 55 | 7.1 | 6 | 1.7 | 58 | 12.0 | 35 | 6.3 |
| Don’t know | 22 | 1.6 | 10 | 1.6 | 12 | 1.6 | 6 | 1.7 | 6 | 1.2 | 10 | 1.8 |
| **What is fascioliasis? *(multiple choices possible)*** | **N= 205** |  | **N= 84** |  | **N= 121** |  | **N= 27** |  | **N= 106** |  | **N= 72** |  |
| A disease affecting skin | 7 | 3.4 | 1 | 1.2 | 6 | 5.0 | 0 | 0.0 | 5 | 4.7 | 2 | 2.8 |
| A disease affecting liver | 158 | 77.1 | 64 | 76.2 | 94 | 77.7 | 18 | 66.7 | 86 | 81.1 | 54 | 75.0 |
| A disease affecting bones | 1 | 0.5 | 0 | 0.0 | 1 | 0.8 | 0 | 0.0 | 1 | 0.9 | 0 | 0.0 |
| A disease affecting heart | 2 | 1 | 0 | 0.0 | 2 | 1.7 | 0 | 0.0 | 1 | 0.9 | 1 | 1.4 |
| Other | 2 | 1 | 0 | 0.0 | 2 | 1.7 | 0 | 0.0 | 1 | 0.9 | 1 | 1.4 |
| Don’t know | 37 | 18.1 | 19 | 22.6 | 18 | 14.9 | 9 | 33.3 | 13 | 12.3 | 15 | 20.8 |
| **In your opinion, how serious a disease is fascioliasis?** | **N= 203** |  | **N= 83** |  | **N= 120** |  | **N= 27** |  | **N= 105** |  | **N= 71** |  |
| Very serious | 41 | 20.2 | 10 | 12.1 | 31 | 25.8 | 2 | 7.4 | 22 | 21.0 | 17 | 23.9 |
| Somewhat serious | 142 | 70.0 | 61 | 73.5 | 81 | 67.5 | 19 | 70.4 | 75 | 71.4 | 48 | 67.6 |
| Not very serious | 3 | 1.5 | 2 | 2.4 | 1 | 0.8 | 1 | 3.7 | 1 | 1.0 | 1 | 1.4 |
| Don’t know | 17 | 8.4 | 10 | 12.1 | 7 | 5.8 | 5 | 18.5 | 7 | 6.7 | 5 | 7.0 |
| **In your opinion, how serious a problem is fascioliasis in your country?** | **N= 204** |  | **N= 84** |  | **N= 120** |  | **N= 27** |  | **N= 105** |  | **N= 72** |  |
| Very serious | 38 | 18.6 | 10 | 11.9 | 28 | 23.3 | 2 | 7.4 | 21.0 | 20.0 | 15 | 20.8 |
| Somewhat serious | 146 | 71.6 | 62 | 73.8 | 84 | 70.0 | 18 | 66.7 | 77.0 | 73.3 | 51 | 70.8 |
| Not very serious | 3 | 1.5 | 2 | 2.4 | 1 | 0.8 | 2 | 7.4 | 1.0 | 1.0 | 0 | 0.0 |
| Don’t know | 17 | 8.3 | 10 | 11.9 | 7 | 5.8 | 5 | 18.5 | 6.0 | 5.7 | 6 | 8.3 |
| **What symptoms does fascioliasis cause? *(multiple choices possible)*** | **N= 205** |  | **N= 84** |  | **N= 121** |  | **N= 27** |  | **N= 106** |  | **N= 72** |  |
| Headache | 32 | 15.6 | 14 | 16.7 | 18 | 14.9 | 1 | 3.7 | 22 | 20.8 | 9 | 12.5 |
| Epilepsy | 5 | 2.4 | 1 | 1.2 | 4 | 3.3 | 0 | 0.0 | 3 | 2.8 | 2 | 2.8 |
| Blurry vision | 6 | 2.9 | 4 | 4.8 | 2 | 1.7 | 0 | 0.0 | 5 | 4.7 | 1 | 1.4 |
| Backache | 6 | 2.9 | 2 | 2.4 | 4 | 3.3 | 1 | 3.7 | 4 | 3.8 | 1 | 1.4 |
| Abdominal pain | 91 | 44.4 | 39 | 46.4 | 52 | 43.0 | 5 | 18.5 | 54 | 50.9 | 32 | 44.4 |
| Fever | 32 | 15.6 | 14 | 16.7 | 18 | 14.9 | 1 | 3.7 | 23 | 21.7 | 8 | 11.1 |
| Itch | 24 | 11.7 | 8 | 9.5 | 16 | 13.2 | 0 | 0.0 | 17 | 16.0 | 7 | 9.7 |
| Rash on skin | 9 | 4.4 | 2 | 2.4 | 7 | 5.8 | 1 | 3.7 | 4 | 3.8 | 4 | 5.6 |
| Jaundice | 1 | 0.5 | 0 | 0.0 | 1 | 0.8 | 0 | 0.0 | 1 | 0.9 | 0 | 0.0 |
| Other | 6 | 2.9 | 1 | 1.2 | 5 | 4.1 | 0 | 0.0 | 3 | 2.8 | 3 | 4.2 |
| Don’t know | 82 | 40.0 | 39 | 46.4 | 43 | 35.5 | 20 | 74.1 | 31 | 29.3 | 31 | 43.1 |
| **What agent is causing fascioliasis? *(multiple choices possible)*** | **N= 205** |  | **N= 84** |  | **N= 121** |  | **N= 27** |  | **N= 106** |  | **N= 72** |  |
| Bad weather | 1 | 0.5 | 1 | 1.2 | 0 | 0.0 | 1 | 3.7 | 0 | 0.0 | 0 | 0.0 |
| Lack of nutritious food | 0 | 0.0 | 0 | 0.0 | 0 | 0.0 | 0 | 0.0 | 0 | 0.0 | 0 | 0.0 |
| You are born with it | 0 | 0.0 | 0 | 0.0 | 0 | 0.0 | 0 | 0.0 | 0 | 0.0 | 0 | 0.0 |
| A virus | 8 | 3.9 | 5 | 6.0 | 3 | 2.5 | 2 | 7.4 | 2 | 1.9 | 4 | 5.6 |
| A parasite | 126 | 61.5 | 52 | 61.9 | 74 | 61.2 | 15 | 55.6 | 68 | 64.2 | 43 | 59.7 |
| A bacteria | 6 | 2.9 | 0 | 0.0 | 6 | 5.0 | 1 | 3.7 | 4 | 3.8 | 1 | 1.4 |
| Other | 1 | 0.5 | 0 | 0.0 | 1 | 0.8 | 0 | 0.0 | 1 | 0.9 | 0 | 0.0 |
| Don’t know | 64 | 31.2 | 26 | 31.0 | 38 | 31.4 | 9 | 33.3 | 30 | 28.3 | 25 | 34.7 |
| **How can a person get fascioliasis? *(multiple choices possible)*** | **N= 204** |  | **N= 83** |  | **N= 121** |  | **N= 26** |  | **N= 106** |  | **N= 72** |  |
| Through consumption infected organs | 17 | 8.3 | 8 | 9.6 | 9 | 7.4 | 4 | 15.4 | 6 | 5.7 | 7 | 9.7 |
| Through consumption contaminated plants/vegetables | 124 | 60.8 | 50 | 60.2 | 74 | 61.2 | 7 | 26.9 | 72 | 67.9 | 45 | 62.5 |
| Through consumption infected meat | 14 | 6.9 | 2 | 2.4 | 12 | 9.9 | 1 | 3.9 | 7 | 6.6 | 6 | 8.3 |
| Through drinking alcohol | 0 | 0.0 | 0 | 0.0 | 0 | 0.0 | 0 | 0.0 | 0 | 0.0 | 0 | 0.0 |
| Through consumption of infected fish | 2 | 1.0 | 0 | 0.0 | 2 | 1.7 | 0 | 0.0 | 0 | 0.0 | 2 | 2.8 |
| Contact with animals | 3 | 1.5 | 1 | 1.2 | 2 | 1.7 | 1 | 3.9 | 2 | 1.9 | 0 | 0.0 |
| Other | 4 | 2.0 | 0 | 0.0 | 4 | 3.3 | 1 | 3.9 | 2 | 1.9 | 1 | 1.4 |
| Don’t know | 62 | 30.4 | 29 | 34.9 | 33 | 27.3 | 13 | 50.0 | 26 | 24.5 | 23 | 31.9 |
| **How can human fascioliasis be prevented? *(multiple choices possible)*** | **N= 205** |  | **N= 84** |  | **N= 121** |  | **N= 27** |  | **N= 106** |  | **N= 72** |  |
| Isolating infected livestock | 4 | 2.0 | 0 | 0.0 | 4 | 3.3 | 0 | 0.0 | 3 | 2.8 | 1 | 1.4 |
| Isolating infected humans | 1 | 0.5 | 0 | 0.0 | 1 | 0.8 | 0 | 0.0 | 0 | 0.0 | 1 | 1.4 |
| Washing water plants | 24 | 11.7 | 6 | 7.1 | 18 | 14.9 | 0 | 0.0 | 14 | 13.2 | 10 | 13.9 |
| Do not eat raw water plants | 87 | 42.4 | 44 | 52.4 | 43 | 35.5 | 2 | 7.4 | 55 | 51.9 | 30 | 41.7 |
| Cooking meat or organs | 37 | 18.1 | 12 | 14.3 | 25 | 20.7 | 7 | 25.9 | 15 | 14.2 | 15 | 20.8 |
| Cooking vegetables | 51 | 24.9 | 16 | 19.1 | 35 | 28.9 | 8 | 29.6 | 22 | 20.8 | 21 | 29.2 |
| Treatment of humans | 4 | 2.0 | 2 | 2.4 | 2 | 1.7 | 1 | 3.7 | 3 | 2.8 | 0 | 0.0 |
| Treatment of animals | 2 | 1.0 | 1 | 1.2 | 1 | 0.8 | 1 | 3.7 | 1 | 0.9 | 0 | 0.0 |
| It cannot be prevented | 0 | 0.0 | 0 | 0.0 | 0 | 0.0 | 0 | 0.0 | 0 | 0.0 | 0 | 0.0 |
| Other | 0 | 0.0 | 0 | 0.0 | 0 | 0.0 | 0 | 0.0 | 0 | 0.0 | 0 | 0.0 |
| Don’t know | 56 | 27.3 | 24 | 28.6 | 32 | 26.5 | 13 | 48.2 | 23 | 21.7 | 20 | 27.8 |
| **In your opinion, who can be infected with human fascioliasis? *(multiple choices possible)*** | **N= 205** |  | **N= 84** |  | **N= 121** |  | **N= 27** |  | **N= 106** |  | **N= 72** |  |
| Only children | 6 | 2.9 | 1 | 1.2 | 5 | 4.1 | 1 | 3.7 | 5 | 4.7 | 0 | 0.0 |
| Only poor people | 0 | 0.0 | 0 | 0.0 | 0 | 0.0 | 0 | 0.0 | 0 | 0.0 | 0 | 0.0 |
| Only homeless people | 1 | 0.5 | 1 | 1.2 | 0 | 0.0 | 1 | 3.7 | 0 | 0.0 | 0 | 0.0 |
| Only elderly people | 6 | 2.9 | 2 | 2.4 | 4 | 3.3 | 2 | 7.4 | 3 | 2.8 | 1 | 1.4 |
| Only men | 3 | 1.5 | 2 | 2.4 | 1 | 0.8 | 0 | 0.0 | 2 | 1.9 | 1 | 1.4 |
| Only women | 0 | 0.0 | 0 | 0.0 | 0 | 0.0 | 0 | 0.0 | 0 | 0.0 | 0 | 0.0 |
| Anybody | 155 | 75.6 | 62 | 73.8 | 93 | 76.9 | 16 | 59.3 | 84 | 79.3 | 55 | 76.4 |
| Other | 1 | 0.5 | 0 | 0.0 | 1 | 0.8 | 0 | 0.0 | 0 | 0.0 | 1 | 1.4 |
| Don’t know | 35 | 17.1 | 17 | 20.2 | 18 | 14.9 | 8 | 29.6 | 13 | 12.3 | 14 | 19.4 |
| **Can fascioliasis be cured? *(multiple choices possible)*** | **N= 205** |  | **N= 84** |  | **N= 121** |  | **N= 27** |  | **N= 106** |  | **N= 72** |  |
| Yes, with herbal medicine | 2 | 1.0 | 1 | 1.2 | 1 | 0.8 | 0 | 0.0 | 1 | 0.9 | 1 | 1.4 |
| Yes, home rest without medicine | 8 | 3.9 | 4 | 4.8 | 4 | 3.3 | 0 | 0.0 | 8 | 7.6 | 0 | 0.0 |
| Yes, specific treatment given by health centre | 161 | 78.5 | 60 | 71.4 | 101 | 83.5 | 15 | 55.6 | 84 | 79.3 | 62 | 86.1 |
| Yes, using another method, specify:___________________ | 0 | 0 | 0 | 0.0 | 0 | 0.0 | 0 | 0.0 | 0 | 0.0 | 0 | 0.0 |
| It cannot be cured | 5 | 2.4 | 2 | 2.4 | 3 | 2.5 | 2 | 7.4 | 2 | 1.9 | 1 | 1.4 |
| Don’t know | 30 | 14.6 | 18 | 21.4 | 12 | 9.9 | 10 | 37.0 | 12 | 11.3 | 8 | 11.1 |
| **Where did you hear about fascioliasis? *(multiple choices possible)*** | **N= 205** |  | **N= 84** |  | **N= 121** |  | **N= 27** |  | **N= 106** |  | **N= 72** |  |
| School | 11 | 5.4 | 7 | 8.3 | 4 | 3.3 | 8 | 29.6 | 3 | 2.8 | 0 | 0.0 |
| Internet | 4 | 2.0 | 3 | 3.6 | 1 | 0.8 | 1 | 3.7 | 2 | 1.9 | 1 | 1.4 |
| Newspapers and magazines | 50 | 24.4 | 22 | 26.2 | 28 | 23.1 | 6 | 22.2 | 31 | 29.3 | 13 | 18.1 |
| Radio | 53 | 25.9 | 25 | 29.8 | 28 | 23.1 | 3 | 11.1 | 27 | 25.5 | 23 | 31.9 |
| TV | 83 | 40.5 | 35 | 41.7 | 48 | 39.7 | 8 | 29.6 | 50 | 47.2 | 25 | 34.7 |
| Billboards/posters | 1 | 0.5 | 1 | 1.2 | 0 | 0.0 | 0 | 0.0 | 1 | 0.9 | 0 | 0.0 |
| Health workers | 69 | 33.7 | 24 | 28.6 | 45 | 37.2 | 5 | 18.5 | 38 | 35.9 | 26 | 36.1 |
| Family, friends, neighbours and colleagues | 27 | 13.2 | 6 | 7.1 | 21 | 17.4 | 2 | 7.4 | 15 | 14.2 | 10 | 13.9 |
| Other | 2 | 1.0 | 0 | 0.0 | 2 | 1.7 | 1 | 3.7 | 0 | 0.0 | 1 | 1.4 |
| Don’t know | 16 | 7.8 | 10 | 11.9 | 6 | 5.0 | 6 | 22.2 | 4 | 3.8 | 6 | 8.3 |
| **Do you know people who have/had fascioliasis? *(multiple choices possible)*** | **N= 205** |  | **N= 84** |  | **N= 121** |  | **N= 27** |  | **N= 106** |  | **N= 72** |  |
| Yes, I have/had fascioliasis | 4 | 2.0 | 0 | 0.0 | 4 | 3.3 | 0 | 0.0 | 1 | 0.9 | 3 | 4.2 |
| Yes, household members | 4 | 2.0 | 1 | 1.2 | 3 | 2.5 | 0 | 0.0 | 3 | 2.8 | 1 | 1.4 |
| Yes, relatives (not from household) | 6 | 2.9 | 1 | 1.2 | 5 | 4.1 | 0 | 0.0 | 4 | 3.8 | 2 | 2.8 |
| Yes, neighbours | 13rv | 6.3 | 2 | 2.4 | 11 | 9.1 | 0 | 0.0 | 6 | 5.7 | 7 | 9.7 |
| Yes, friends or colleagues | 1 | 0.5 | 0 | 0.0 | 1 | 0.8 | 0 | 0.0 | 0 | 0.0 | 1 | 1.4 |
| Yes, other people | 0 | 0.0 | 0 | 0.0 | 0 | 0.0 | 0 | 0.0 | 0 | 0.0 | 0 | 0.0 |
| No | 90 | 43.9 | 37 | 44.1 | 53 | 43.8 | 12 | 44.4 | 49 | 46.2 | 29 | 40.3 |
| Don’t know | 87 | 42.4 | 43 | 51.2 | 44 | 36.4 | 15 | 55.6 | 42 | 39.6 | 30 | 41.7 |
| **If you have or had fascioliasis, at what point did you go to the health facility?** | **N= 4** |  | **N= 0** |  | **N= 4** |  | **N= 0** |  | **N= 1** |  | **N= 3** |  |
| When treatment on my own didn’t work | 0 | 0.0 | 0 | 0.0 | 0 | 0.0 | 0 | 0.0 | 0 | 0.0 | 0 | 0.0 |
| As soon as I realized that my symptoms might be related with fascioliasis | 0 | 0.0 | 0 | 0.0 | 0 | 0.0 | 0 | 0.0 | 0 | 0.0 | 0 | 0.0 |
| As soon as I was feeling ill | 3 | 1.5 | 0 | 0.0 | 3 | 75.0 | 0 | 0.0 | 1 | 100.0 | 2 | 66.7 |
| I did not go to the health facility | 1 | 0.5 | 0 | 0.0 | 1 | 25.0 | 0 | 0.0 | 0 | 0.0 | 1 | 33.3 |
| Other | 0 | 0.0 | 0 | 0.0 | 0 | 0.0 | 0 | 0.0 | 0 | 0.0 | 0 | 0.0 |
| Don’t know | 0 | 0.0 | 0 | 0.0 | 0 | 0.0 | 0 | 0.0 | 0 | 0.0 | 0 | 0.0 |

**Table B. Attitudes and practices related to fascioliasis among the participants in Dong Thanh commune**

| **Question** | **Total** | | **Gender** | | | | **Age (years)** | | | | | |
| --- | --- | --- | --- | --- | --- | --- | --- | --- | --- | --- | --- | --- |
|  |  |  | **Male** | | **Female** | | **<18** | | **18-49** | | **>=50** | |
|  | **n** | **%** | **n** | **%** | **n** | **%** | **n** | **%** | **n** | **%** | **n** | **%** |
| **Do you think you could get fascioliasis?** | **N= 204** |  | **N= 84** |  | **N= 120** |  | **N= 27** |  | **N= 105** |  | **N= 72** |  |
| Yes, because | 119 | 58.3 | 45 | 53.6 | 74 | 61.7 | 13 | 48.2 | 66 | 62.9 | 40 | 55.6 |
| No, because | 13 | 6.4 | 6 | 7.1 | 7 | 5.8 | 1 | 3.7 | 6 | 5.7 | 6 | 8.3 |
| Don’t know | 72 | 35.3 | 33 | 39.3 | 39 | 32.5 | 13 | 48.2 | 33 | 31.4 | 26 | 36.1 |
| **What would be your reaction if you were to find out that you have fascioliasis? *(multiple choices possible)*** | **N= 205** |  | **N= 84** |  | **N= 121** |  | **N= 27** |  | **N= 106** |  | **N= 72** |  |
| Normal | 10 | 4.9 | 6 | 7.1 | 4 | 3.3 | 0 | 0.0 | 7 | 6.6 | 3 | 4.2 |
| Fear | 172 | 83.9 | 64 | 76.2 | 108 | 89.3 | 17 | 63.0 | 94 | 88.7 | 61 | 84.7 |
| Surprise | 4 | 2.0 | 2 | 2.4 | 2 | 1.7 | 2 | 7.4 | 1 | 0.9 | 1 | 1.4 |
| Sadness or hopelessness | 1 | 0.5 | 0 | 0.0 | 1 | 0.8 | 0 | 0.0 | 0 | 0.0 | 1 | 1.4 |
| Shame | 0 | 0.0 | 0 | 0.0 | 0 | 0.0 | 0 | 0.0 | 0 | 0.0 | 0 | 0.0 |
| Other | 1 | 0.5 | 0 | 0.0 | 1 | 0.8 | 0 | 0.0 | 1 | 0.9 | 0 | 0.0 |
| Don’t know | 18 | 8.8 | 12 | 14.3 | 6 | 5.0 | 8 | 29.6 | 3 | 2.8 | 7 | 9.7 |
| **Where do you usually go if you are sick, or to treat a general health problem? *(multiple choices possible)*** | **N= 1,398** |  | **N= 626** |  | **N= 772** |  | **N= 355** |  | **N= 485** |  | **N= 558** |  |
| Go to health facility | 1340 | 95.9 | 599 | 95.7 | 741 | 96.0 | 304 | 85.6 | 481 | 99.2 | 555 | 99.5 |
| Go to pharmacy | 46 | 3.3 | 12 | 1.9 | 34 | 4.4 | 9 | 2.5 | 20 | 4.1 | 17 | 3.1 |
| Go to traditional healer | 0 | 0.0 | 0 | 0.0 | 0 | 0.0 | 0 | 0.0 | 0 | 0.0 | 0 | 0.0 |
| Pursue other self-treatment options | 0 | 0.0 | 0 | 0.0 | 0 | 0.0 | 0 | 0.0 | 0 | 0.0 | 0 | 0.0 |
| Nowhere, rest at home | 2 | 0.1 | 2 | 0.3 | 0 | 0.0 | 2 | 0.6 | 0 | 0.0 | 0 | 0.0 |
| Other | 0 | 0.0 | 0 | 0.0 | 0 | 0.0 | 0 | 0.0 | 0 | 0.0 | 0 | 0.0 |
| Don’t know | 46 | 3.3 | 21 | 3.4 | 25 | 3.2 | 43 | 12.1 | 1 | 0.2 | 2 | 0.4 |
| **If you would not go to the health facility, what is the reason? *(multiple choices possible)*** | **N= 58** |  | **N= 27** |  | **N= 31** |  | **N= 51** |  | **N= 4** |  | **N= 3** |  |
| No need | 2 | 3.5 | 2 | 7.4 | 0 | 0.0 | 2 | 3.9 | 0 | 0.0 | 0 | 0.0 |
| Not sure where to go | 2 | 3.5 | 0 | 0.0 | 2 | 6.5 | 2 | 3.9 | 0 | 0.0 | 0 | 0.0 |
| Cost | 1 | 1.7 | 1 | 3.7 | 0 | 0.0 | 1 | 2.0 | 0 | 0.0 | 0 | 0.0 |
| Difficulties with transportation/distance to clinic | 3 | 5.2 | 0 | 0.0 | 3 | 9.7 | 2 | 3.9 | 0 | 0.0 | 1 | 33.3 |
| Do not trust medical workers | 0 | 0.0 | 0 | 0.0 | 0 | 0.0 | 0 | 0.0 | 0 | 0.0 | 0 | 0.0 |
| Do not like attitude of medical workers | 0 | 0.0 | 0 | 0.0 | 0 | 0.0 | 0 | 0.0 | 0 | 0.0 | 0 | 0.0 |
| Cannot leave work (overlapping work hours with medical facility working hours) | 2 | 3.5 | 1 | 3.7 | 1 | 3.2 | 0 | 0.0 | 2 | 50.0 | 0 | 0.0 |
| Do not want to find out something is really wrong | 0 | 0.0 | 0 | 0.0 | 0 | 0.0 | 0 | 0.0 | 0 | 0.0 | 0 | 0.0 |
| Other | 0 | 0.0 | 0 | 0.0 | 0 | 0.0 | 0 | 0.0 | 0 | 0.0 | 0 | 0.0 |
| Don’t know | 37 | 63.8 | 18 | 66.7 | 19 | 61.3 | 34 | 66.7 | 1 | 25.0 | 2 | 66.7 |
| **How often do you generally seek health care at a clinic or hospital** | **N= 1,340** |  | **N= 599** |  | **N= 741** |  | **N= 304** |  | **N= 481** |  | **N= 555** |  |
| Twice a year or more | 612 | 45.7 | 262 | 43.7 | 350 | 47.2 | 119 | 39.1 | 221 | 46.0 | 272 | 49.0 |
| Once per year | 521 | 38.9 | 230 | 38.4 | 291 | 39.3 | 114 | 37.5 | 189 | 39.3 | 218 | 39.3 |
| Less than once a year | 183 | 13.7 | 96 | 16.0 | 87 | 11.7 | 50 | 16.5 | 67 | 13.9 | 66 | 11.9 |
| Don’t know | 24 | 1.8 | 12 | 2.0 | 12 | 1.6 | 20 | 6.6 | 4 | 0.8 | 0 | 0.0 |
| **Do you consume one of these plants? *(pictures of vegetables are shown, Fig 2)*** | **N= 1,392** |  | **N= 623** |  | **N= 769** |  | **N= 352** |  | **N= 484** |  | **N= 556** |  |
| Yes | 1265 | 90.9 | 554 | 88.9 | 711 | 92.5 | 239 | 67.9 | 482 | 99.6 | 544 | 97.8 |
| No | 101 | 7.3 | 52 | 8.4 | 49 | 6.4 | 89 | 25.3 | 1 | 0.2 | 11 | 2.0 |
| Don’t know | 26 | 1.9 | 17 | 2.7 | 9 | 1.2 | 24 | 6.8 | 1 | 0.2 | 1 | 0.2 |
| **Do you prepare one of these plants at home? *(multiple choices possible)*** | **N= 1,391** |  | **N= 622** |  | **N= 769** |  | **N= 352** |  | **N= 484** |  | **N= 555** |  |
| Yes | 1161 | 83.5 | 474 | 76.2 | 687 | 89.3 | 190 | 54.0 | 458 | 94.6 | 513 | 92.4 |
| No | 204 | 14.7 | 133 | 21.4 | 71 | 9.2 | 138 | 39.2 | 25 | 5.2 | 41 | 7.4 |
| Don’t know | 26 | 1.9 | 15 | 2.4 | 11 | 1.4 | 24 | 6.8 | 1 | 0.2 | 1 | 0.2 |
| **Which of the following plants do you consume raw? *(multiple choices possible)*** | **N= 1,168** |  | **N= 478** |  | **N= 690** |  | **N= 193** |  | **N= 459** |  | **N= 516** |  |
| Water spinach, water morning glory | 75 | 6.4 | 40 | 8.4 | 35 | 5.1 | 7 | 3.6 | 41 | 8.9 | 27 | 5.2 |
| Water cress | 18 | 1.5 | 11 | 2.3 | 7 | 1.0 | 0 | 0.0 | 9 | 2.0 | 9 | 1.7 |
| Rice paddy herb | 118 | 10.1 | 67 | 14.0 | 51 | 7.4 | 5 | 2.6 | 57 | 12.4 | 56 | 10.9 |
| Lettuce | 1049 | 89.8 | 429 | 89.8 | 620 | 89.9 | 142 | 73.6 | 435 | 94.8 | 472 | 91.5 |
| Sweet Cabbage | 40 | 3.4 | 23 | 4.8 | 17 | 2.5 | 8 | 4.2 | 19 | 4.1 | 13 | 2.5 |
| Lotus | 5 | 0.4 | 5 | 1.1 | 0 | 0.0 | 1 | 0.5 | 2 | 0.4 | 2 | 0.4 |
| Fish mint, lettuce mint | 661 | 56.6 | 262 | 54.8 | 399 | 57.8 | 51 | 26.4 | 302 | 65.8 | 308 | 59.7 |
| Water dropwort | 16 | 1.4 | 8 | 1.7 | 8 | 1.2 | 0 | 0.0 | 8 | 1.7 | 8 | 1.6 |
| Other non-water plant | 88 | 7.5 | 36 | 7.5 | 52 | 7.5 | 9 | 4.7 | 45 | 9.8 | 34 | 6.6 |
| Other | 0 | 0.0 | 0 | 0.0 | 0 | 0.0 | 0 | 0.0 | 0 | 0.0 | 0 | 0.0 |
| I don’t consume raw plants/vegetables | 77 | 6.6 | 36 | 7.5 | 41 | 5.9 | 41 | 21.2 | 9 | 2.0 | 27 | 5.2 |
| Don’t know | 8 | 0.7 | 3 | 0.6 | 5 | 0.7 | 7 | 3.6 | 0 | 0.0 | 1 | 0.2 |
| **If you consume these raw, how often? (last year)** | **N= 1,168** |  | **N= 478** |  | **N= 690** |  | **N= 193** |  | **N= 459** |  | **N= 516** |  |
| Daily | 22 | 1.9 | 11 | 2.3 | 11 | 1.6 | 1 | 0.5 | 7 | 1.5 | 14 | 2.7 |
| At least once a week | 690 | 59.1 | 300 | 62.8 | 390 | 56.5 | 86 | 44.6 | 294 | 64.1 | 310 | 60.1 |
| At least once a month | 335 | 28.7 | 112 | 23.4 | 223 | 32.3 | 50 | 25.9 | 137 | 29.9 | 148 | 28.7 |
| At least once a year | 41 | 3.5 | 16 | 3.4 | 25 | 3.6 | 8 | 4.2 | 12 | 2.6 | 21 | 4.1 |
| Don’t know | 32 | 2.7 | 17 | 3.6 | 15 | 2.2 | 24 | 12.4 | 3 | 0.7 | 5 | 1.0 |
| **If you prepare these at home (either raw or cooked), how often? (last year)** | **N= 1,168** |  | **N= 478** |  | **N= 690** |  | **N= 193** |  | **N= 459** |  | **N= 516** |  |
| Daily | 395 | 33.8 | 160 | 33.5 | 235 | 34.1 | 63 | 32.6 | 144 | 31.4 | 188 | 36.4 |
| At least once a week | 473 | 40.5 | 208 | 43.5 | 265 | 38.4 | 67 | 34.7 | 211 | 46.0 | 195 | 37.8 |
| At least once a month | 228 | 19.5 | 78 | 16.3 | 150 | 21.7 | 33 | 17.1 | 87 | 19.0 | 108 | 20.9 |
| At least once a year | 32 | 2.7 | 13 | 2.7 | 19 | 2.8 | 3 | 1.6 | 12 | 2.6 | 17 | 3.3 |
| Don’t know | 38 | 3.3 | 18 | 3.8 | 20 | 2.9 | 25 | 13.0 | 5 | 1.1 | 8 | 1.6 |
| **Where do you mainly consume these plants?** | **N= 1,168** |  | **N= 478** |  | **N= 690** |  | **N= 193** |  | **N= 459** |  | **N= 516** |  |
| At home | 1119 | 95.8 | 455 | 95.2 | 664 | 96.2 | 177 | 91.7 | 441 | 96.1 | 501 | 97.1 |
| At home of other people in same village | 5 | 0.4 | 3 | 0.6 | 2 | 0.3 | 1 | 0.5 | 1 | 0.2 | 3 | 0.6 |
| At home of other people in different village | 1 | 0.1 | 0 | 0.0 | 1 | 0.1 | 0 | 0.0 | 1 | 0.2 | 0 | 0.0 |
| At the market | 44 | 3.8 | 23 | 4.8 | 21 | 3.0 | 5 | 2.6 | 20 | 4.4 | 19 | 3.7 |
| In a restaurant | 6 | 0.5 | 3 | 0.6 | 3 | 0.4 | 0 | 0.0 | 3 | 0.7 | 3 | 0.6 |
| Other | 0 | 0.0 | 0 | 0.0 | 0 | 0.0 | 0 | 0.0 | 0 | 0.0 | 0 | 0.0 |
| Don’t know | 12 | 1.0 | 4 | 0.8 | 8 | 1.2 | 11 | 5.7 | 0 | 0.0 | 1 | 0.2 |
| **Do you ever consume herbal drinks?** | **N= 1,398** |  | **N= 626** |  | **N= 772** |  | **N= 355** |  | **N= 485** |  | **N= 558** |  |
| Yes, tea | 636 | 45.5 | 311 | 49.7 | 325 | 42.1 | 37 | 10.4 | 243 | 50.1 | 356 | 63.8 |
| Yes, others | 3 | 0.2 | 2 | 0.3 | 1 | 0.1 | 0 | 0.0 | 3 | 0.6 | 0 | 0.0 |
| No | 745 | 53.3 | 307 | 49.0 | 438 | 56.7 | 304 | 85.6 | 239 | 49.3 | 202 | 36.2 |
| Don’t know | 15 | 1.1 | 8 | 1.3 | 7 | 0.9 | 13 | 3.7 | 1 | 0.2 | 1 | 0.2 |
| **Do you ever chew on leaves, grass, or other plants you find outdoors?** | **N= 1,398** |  | **N= 626** |  | **N= 772** |  | **N= 355** |  | **N= 485** |  | **N= 558** |  |
| Yes | 45 | 3.2 | 21 | 3.4 | 24 | 3.1 | 4 | 1.1 | 14 | 2.9 | 27 | 4.8 |
| No | 1336 | 95.6 | 597 | 95.4 | 739 | 95.7 | 335 | 94.4 | 470 | 96.9 | 531 | 95.2 |
| Don’t know | 17 | 1.2 | 8 | 1.3 | 9 | 1.2 | 16 | 4.5 | 1 | 0.2 | 0 | 0.0 |
| **Do you ever consume water chestnut?** | **N= 1,392** |  | **N= 624** |  | **N= 768** |  | **N= 353** |  | **N= 483** |  | **N= 556** |  |
| Yes | 3 | 0.2 | 1 | 0.2 | 2 | 0.3 | 1 | 0.3 | 2 | 0.4 | 0 | 0.0 |
| No | 1077 | 77.4 | 486 | 77.9 | 591 | 77.0 | 272 | 77.1 | 380 | 78.7 | 425 | 76.4 |
| Don’t know | 312 | 22.4 | 137 | 22.0 | 175 | 22.8 | 80 | 22.7 | 101 | 20.9 | 131 | 23.6 |

## Household practices relating to fasciolosis in north-central Vietnam

**Table C. Socio-demographic characteristics of studied participants from household questionnaire in Dong Thanh commune.**

| **Variable** | **Category** | **n** | **%** |
| --- | --- | --- | --- |
| **Role in the household (N=620)** | Household head | 401 | 64.7 |
|  | Other | 219 | 35.3 |
| **Gender (N=621)** | Male | 316 | 50.9 |
|  | Female | 305 | 49.1 |
| **Occupation (N=620)** | Farmer | 525 | 84.7 |
|  | Worker | 43 | 6.9 |
|  | Civil servant/ government, cadre | 24 | 3.9 |
|  | Other | 28 | 4.5 |
| **Marital status (N=621)** | Married | 597 | 96.1 |
|  | Single | 13 | 2.1 |
|  | Widowed | 11 | 1.8 |
| **Education (N=621)** | Did not go to school | 3 | 0.5 |
|  | Primary school | 19 | 3.1 |
|  | Secondary school | 408 | 65.7 |
|  | High school | 162 | 26.1 |
|  | University or higher | 29 | 4.7 |

**Table D. Water and sanitation practices in Dong Thanh commune, Vietnam**

| **Question** | **n** | **%** |
| --- | --- | --- |
| **What is the source of drinking water for members of your household? *(indicate most important one(s))*** | **N= 621** |  |
| Piped water into dwelling | 1 | 0.2 |
| Tube well/borehole | 241 | 38.8 |
| Protected dug well | 171 | 27.5 |
| Rainwater collection | 239 | 38.5 |
| Bottled water | 0 | 0.0 |
| Surface water | 5 | 0.8 |
| Other | 0 | 0.0 |
| Don’t know | 0 | 0.0 |
| **What is the source of water used by your household for other purposes, such as washing vegetables, cooking and hand washing? *(indicate most important one(s))*** | **N= 621** |  |
| Piped water into dwelling | 0 | 0.0 |
| Tube well/ borehole | 339 | 54.6 |
| Protected dug well | 234 | 37.7 |
| Rainwater collection | 59 | 9.5 |
| Bottled water | 2 | 0.3 |
| Surface water | 5 | 0.8 |
| Other | 0 | 0.0 |
| Don’t know | 0 | 0.0 |
| **Do you treat your water in any way to make it safer to drink?** | **N= 619** |  |
| Yes | 613 | 98.7 |
| No | 6 | 1.0 |
| Don’t know | 0 | 0.0 |
| **How do you treat the water to make it safer to drink? *(indicate most important one(s))*** | **N= 615** |  |
| Boil | 115 | 18.7 |
| Using a chemical | 0 | 0.0 |
| Use filter, water machine | 501 | 81.5 |
| Use ceramic filter | 37 | 6.0 |
| Let it stand and settle | 1 | 0.2 |
| Other | 0 | 0.0 |
| Don’t know | 0 | 0.0 |
| **What kind of toilet facility do members of your household use? *(indicate most important one(s))*** | **N= 621** |  |
| Flush / pour flush | 450 | 72.5 |
| Composting toilet (single tank) | 19 | 3.1 |
| Composting toilet (double tank) | 134 | 21.6 |
| Hanging toilet | 0 | 0.0 |
| Dug pit latrine | 18 | 2.9 |
| Bush or field | 1 | 0.2 |
| Other | 0 | 0.0 |
| Don’t know | 0 | 0.0 |
| No toilet | 1 | 0.2 |
| **Where does wastewater from your toilet go? *(indicate most important one(s))*** | **N= 619** |  |
| Piped sewer system | 29 | 4.7 |
| Septic tank | 454 | 73.3 |
| Pond/lake/canal | 7 | 1.1 |
| Composting latrine | 122 | 19.7 |
| Other | 2 | 0.3 |
| Don’t know | 1 | 0.2 |
| **Is the toilet facility shared with other households?** | **N= 617** |  |
| Yes | 7 | 1.1 |
| No | 609 | 98.7 |
| Don’t know | 1 | 0.2 |

**Table E. Crop and livestock management practices of households in the Dong Thanh commune, Vietnam**

| **Question** | **n** | **%** |
| --- | --- | --- |
| **Do you or any of your household currently own any agriculture parcels exclusively or joint with someone else?** | **N= 621** |  |
| Yes | 588 | 94.7 |
| No | 33 | 5.3 |
| Don’t know | 0 | 0.0 |
| **Do you or anyone from your household work on agriculture parcels**? | **N= 31** |  |
| Yes | 4 | 12.9 |
| No | 27 | 87.1 |
| Don’t know | 0 | 0.0 |
| **What is the primary use of this parcel?** | **N= 585** |  |
| Livestock | 7 | 1.2 |
| Crops | 411 | 70.3 |
| Livestock and crops | 167 | 28.6 |
| **What crops are cultivated on this parcel? *(indicate most important one(s))*** | **N= 587** |  |
| Rice | 565 | 96.3 |
| Water plants | 19 | 3.2 |
| Sweet potato/potato | 86 | 14.7 |
| Flower | 2 | 0.3 |
| Groundnuts | 18 | 3.1 |
| Corn | 180 | 30.7 |
| Bean | 22 | 3.8 |
| Fruit | 17 | 2.9 |
| Other | 20 | 3.4 |
| Don’t know | 0 | 0.0 |
| **Do you or any member of your household currently own any livestock exclusively or jointly with someone else?** | **N= 584** |  |
| Yes | 567 | 96.4 |
| No | 21 | 3.6 |
| Don’t know | 0 | 0.0 |
| **What are the feed sources for your livestock? *(indicate most important one(s))*** | **N= 573** |  |
| Cut and carry, grown in or near waterbodies | 29 | 5.1 |
| Cut and carry, grown elsewhere | 93 | 16.2 |
| Tethering | 23 | 4.0 |
| Factory product | 93 | 16.2 |
| Free roaming/grazing in or near waterbodies | 3 | 0.5 |
| Free roaming/ grazing elsewhere | 17 | 3.0 |
| Other processed feed source | 330 | 57.6 |
| Other | 2 | 0.4 |
| Don’t know | 6 | 1.1 |
| **What is the purpose of your livestock? *(indicate most important one(s))*** | **N= 573** |  |
| Dairy | 0 | 0.0 |
| Meat | 551 | 96.2 |
| Skin | 0 | 0.0 |
| Draft power | 32 | 5.6 |
| Sale | 21 | 3.7 |
| Other | 1 | 0.2 |
| Don’t know | 4 | 0.7 |
| **Do your livestock come in or near waterbodies where vegetables for human consumption are being grown?** | **N= 567** |  |
| Often | 40 | 7.1 |
| Sometimes | 107 | 18.9 |
| Never | 281 | 49.6 |
| Don’t know | 139 | 24.5 |
| **Does your livestock come in or near water supply for crop water irrigation?** | **N= 567** |  |
| Often | 12 | 2.1 |
| Sometimes | 184 | 32.5 |
| Never | 233 | 41.1 |
| Don’t know | 138 | 24.3 |
| **What is the source of drinking-water for your livestock? *(indicate most important one(s))*** | **N= 573** |  |
| Piped water into dwelling | 1 | 0.2 |
| Tube well/ borehole | 297 | 51.8 |
| Protected dug well | 201 | 35.1 |
| Rainwater collection | 47 | 8.2 |
| Surface water | 14 | 2.4 |
| Other | 0 | 0.0 |
| Don’t know | 15 | 2.6 |
| **Do you use manure of cattle/buffalo/goat/horse/pig/human as fertiliser of your parcel?** | **N= 588** |  |
| Yes | 395 | 67.2 |
| No | 191 | 32.5 |
| Don’t know | 2 | 0.3 |
| **How do you treat manure before the use as fertiliser?** | **N= 395** |  |
| No treatment | 7 | 1.8 |
| Composting before use | 388 | 98.2 |
| Don’t know | 0 | 0.0 |
| Other | 0 | 0.0 |
| **Did you use pesticides on your parcel the last year?** | **N= 587** |  |
| Yes | 469 | 79.9 |
| No | 56 | 9.5 |
| Don’t know | 62 | 10.6 |

**Table F. Culinary practices among the households in the Dong Thanh commune**

| **Question** | **n** | **%** |
| --- | --- | --- |
| **Does your household consume one of these plants? *(pictures of vegetables are shown, Fig 2)*** | **N= 620** |  |
| Yes | 608 | 98.1 |
| No | 12 | 1.9 |
| Don’t know | 0 | 0.0 |
| **Does your household prepare one of these plants at home? Either raw or cooked? *(pictures of vegetables are shown, Fig 2)*** | **N= 618** |  |
| Yes | 606 | 98.1 |
| No | 12 | 1.9 |
| Don’t know | 0 | 0.0 |
| **Which of the following plants does your household consume raw? *(pictures of vegetables are shown, Fig 2) (indicate most important one(s))*** | **N=617** |  |
| Water spinach, water morning glory | 35 | 5.7 |
| Water cress | 17 | 2.8 |
| Rice Paddy Herb | 83 | 13.4 |
| Lettuce | 569 | 92.1 |
| Sweet Cabbage | 14 | 2.3 |
| Lotus | 3 | 0.5 |
| Fish mint, lettuce mint | 399 | 64.6 |
| Water dropwort | 5 | 0.8 |
| Other non-water plants | 36 | 5.8 |
| Other | 0 | 0.0 |
| Don’t know | 0 | 0.0 |
| My household does not consume raw plants/vegetables | 28 | 4.5 |
| **Which of the following plants does your household consume cooked? *(indicate most important one(s))*** | **N=617** |  |
| Water spinach, water morning glory | 588 | 95.3 |
| Water cress | 72 | 11.7 |
| Rice Paddy Herb | 75 | 12.2 |
| Lettuce | 14 | 2.3 |
| Sweet Cabbage | 528 | 85.6 |
| Lotus | 0 | 0.0 |
| Fish mint, lettuce mint | 4 | 0.7 |
| Water dropwort | 176 | 28.5 |
| Other non-water plants | 44 | 7.1 |
| Other | 0 | 0.0 |
| Don’t know | 0 | 0.0 |
| My household does not consume cooked plants/vegetables | 0 | 0.0 |
| **Does your household wash these plants before use?** | **N= 610** |  |
| Yes | 608 | 99.7 |
| No | 2 | 0.3 |
| Don’t know | 0 | 0.0 |
| **How does your household wash these plants? *(indicate most important one(s))*** | **N= 617** |  |
| With water | 367 | 59.5 |
| With water + vinegar | 8 | 1.3 |
| With water + salt | 250 | 40.5 |
| Other | 0 | 0.0 |
| Don’t know | 1 | 0.2 |
| **Where does your households obtain these plants? *(indicate most important one(s))*** | **N= 617** |  |
| Local market | 108 | 17.5 |
| Cultivated from own parcel | 522 | 84.6 |
| Cultivated from another parcel | 2 | 0.3 |
| Super market | 0 | 0.0 |
| Restaurant | 0 | 0.0 |
| Other | 0 | 0.0 |
| Don’t know | 0 | 0.0 |
